# The *Drosophila* Transcription Factor Dimmed Affects Neuronal Growth and Differentiation in Multiple Ways Depending on Neuron Type and Developmental Stage

**DOI:** 10.3389/fnmol.2016.00097

**Published:** 2016-10-13

**Authors:** Yiting Liu, Jiangnan Luo, Dick R. Nässel

**Affiliations:** Department of Zoology, Stockholm UniversityStockholm, Sweden

**Keywords:** bHLH transcription factor, insulin-like peptide, insulin receptor, apoptosis, neuroendocrine cells, neuropeptide

## Abstract

Growth of postmitotic neurons occurs during different stages of development, including metamorphosis, and may also be part of neuronal plasticity and regeneration. Recently we showed that growth of post-mitotic neuroendocrine cells expressing the basic helix loop helix (bHLH) transcription factor Dimmed (Dimm) in *Drosophila* could be regulated by insulin/IGF signaling and the insulin receptor (dInR). Dimm is also known to confer a secretory phenotype to neuroendocrine cells and can be part of a combinatorial code specifying terminal differentiation in peptidergic neurons. To further understand the mechanisms of *Dimm* function we ectopically expressed Dimm or Dimm together with dInR in a wide range of Dimm positive and Dimm negative peptidergic neurons, sensory neurons, interneurons, motor neurons, and gut endocrine cells. We provide further evidence that dInR mediated cell growth occurs in a Dimm dependent manner and that one source of insulin-like peptide (DILP) for dInR mediated cell growth in the CNS is DILP6 from glial cells. Expressing both Dimm and dInR in Dimm negative neurons induced growth of cell bodies, whereas dInR alone did not. We also found that Dimm alone can regulate cell growth depending on specific cell type. This may be explained by the finding that the dInR is a direct target of Dimm. Conditional gene targeting experiments showed that Dimm alone could affect cell growth in certain neuron types during metamorphosis or in the adult stage. Another important finding was that ectopic Dimm inhibits apoptosis of several types of neurons normally destined for programmed cell death (PCD). Taken together our results suggest that Dimm plays multiple transcriptional roles at different developmental stages in a cell type-specific manner. In some cell types ectopic Dimm may act together with resident combinatorial code transcription factors and affect terminal differentiation, as well as act in transcriptional networks that participate in long term maintenance of neurons which might lead to blocked apoptosis.

## Introduction

Growth of neurons occurs during different stages of development and may also be part of neuron plasticity and regeneration. Size scaling of neurons is another important aspect of differential growth resulting in large motor neurons and neuroendocrine cells. Recently it was shown that growth of post-mitotic neuroendocrine cells in *Drosophila* is regulated by insulin/IGF signaling (IIS), combined with nutrient dependent target of rapamycin (TOR) signaling (Luo et al., 2013; Gu et al., 2014). Manipulations of insulin receptor (dInR) expression in different neuron types was only found effective in those that are known to express the basic helix-loop-helix (bHLH) transcription factor *Dimmed (Dimm)* (Luo et al., 2013). *Dimm* regulates genes that confer a prominent secretory phenotype to neurons and neuroendocrine cells (Hewes et al., 2003, 2006; Park et al., 2011), but it also acts with other transcription factors in sets of combinatorial codes to specify neurons during terminal differentiation (Allan et al., 2005; Baumgardt et al., 2007; Benito-Sipos et al., 2010). In the larval nervous system of *Drosophila* there are about 300 *Dimm* expressing neurons most of which have been classified as peptidergic neuroendocrine cells (Park et al., 2008b). Many of these neurons have relatively large cell bodies and are likely to display high secretory capacity. It has been suggested that *Dimm* expressing neurons produce large quantities of neuropeptides and peptide hormones that are released intermittently in bulk (Park et al., 2008b). In contrast, several types of neuropeptides are produced in *Dimm*-negative interneurons, many of which have small cell bodies and axon terminations (Park et al., 2008b). It is possible that in these neurons the neuropeptide is used as a co-transmitter more locally and, thus, less peptide production is required.

In a previous study (Luo et al., 2013) we proposed that the dInR-mediated growth regulation of *Dimm*-positive neurons is part of a mechanism to ensure plasticity in growth of the neurons under different nutritional conditions. Another paper reporting dInR and TOR mediated growth regulation (Gu et al., 2014) did not show the link to *Dimm*, but demonstrated that in a specific set of peptidergic neurons the growth-regulation was associated with dynamics of neuron morphology during metamorphosis. These authors analyzed a set of Dimm positive neurons that produce the neuropeptides crustacean cardioactive peptide (CCAP) and bursicon, not included in our study. In contrast to our findings on several other neuron types, it was observed that the size of the CCAP/Bursicon neurons was not affected by dInR manipulations in the larval stages, but seen only later (Gu et al., 2014). Thus, the CCAP neurons may respond differently to IIS than the Dimm-positive neuron types in our study, suggesting variability in Dimm function. Therefore, a screen of dInR-mediated growth in further Dimm-positive neurons may be useful to unravel roles of Dimm in cell differentiation.

Genome wide analysis identified a number of direct Dimm binding targets that are associated with key roles in determination of important properties of neuroendocrine cells, linked to large dense core vesicles and the secretory pathway (Hadžić et al., 2015). As a consequence of this ectopic Dimm is able to transform Dimm negative neurons toward a neuroendocrine phenotype. It was shown that *Drosophila* photoreceptors respond to ectopic Dimm expression by altered morphology, loss of histaminergic phenotype, and production of dense cored vesicles (Hamanaka et al., 2010). Another study showed that ectopic Dimm expression in aCC motor neurons causes accumulation of peptidergic vesicles in axonal boutons and transformation of these boutons from type Ib toward a morphology more similar to peptidergic type III boutons, that display enhanced capture of presynaptic dense core vesicle (Bulgari et al., 2014). One of the Dimm binding targets identified by chromatin immunoprecipitation is the dInR (Hadžić et al., 2015). This encouraged us to further investigate the link between Dimm and the dInR, and the possibility that ectopic Dimm generates neurons responding to IIS by increasing their size.

Here, we ask whether growth of Dimm-negative neurons can be triggered by IIS if Dimm is ectopically expressed. We investigate growth and morphology of Dimm positive and Dimm negative neurons of several types to determine effects of targeted expression of Dimm alone, as well as Dimm combined with the dInR. Dimm negative neurons respond to combined Dimm and dInR expression by growth, whereas Dimm positive neurons display variable responses, depending on neuron type and developmental stage. In some cases Dimm alone triggers growth in Dimm negative neurons and interestingly ectopic Dimm causes block of cell death in several types of neurons that normally undergo programmed apoptosis during or just after metamorphosis. Thus, our study reveals a number of actions of ectopic Dimm that link to dInR responsiveness and growth, but also possible roles of Dimm as a transcriptional code specifying neuron phenotype and partaking in adult neuron maintenance.

## Materials and methods

### Fly lines and husbandry

As control flies in all experiments we used the *Drosophila melanogaster w*^*1118*^ strain from the Bloomington *Drosophila* Stock Center (BDSC), Bloomington, IN. Adult male flies were used for experiments, unless otherwise stated. We utilized *w; Lk*-Gal4 (gift from P. Herrero, Madrid, Spain; de Haro et al., 2010), *yw; Crz*-Gal4 and *yw; Pdf* -Gal4 (from J.H. Park, Knoxville, TN; Choi et al., 2008), *w; Ccap*-Gal4 (from John Ewer, Valpariso, Chile, provided by C. Wegener, Würzburg, Germany; Park et al., 2003), *w; Eh*-Gal4 (BDSC) (McNabb et al., 1997), *w; Kurs6*-Gal4 (from G. Korge, Berlin, Germany; Siegmund and Korge, 2001), *yw; Ptth*-Gal4; UAS-GFP (from K. Rewitz, Copenhagen, Denmark; McBrayer et al., 2007), *w; Trh*-Gal4 (from E. A. Kravitz, Boston, MA; Alekseyenko et al., 2010), *w; Sco/Cyo; RRa*-Gal4; *mcd8-gfp* (from Richard Baines, Manchester, UK; Fujioka et al., 2003), *w; longGMR*-Gal4 and *GMR-Gal4*; UAS-*dimm* (from D. Park, St Louis, MO; Hamanaka et al., 2010), *w; Repo*-Gal4 (from BDSC), *yw*; UAS-*dInR* (from BDSC), *w; “ITP*-Gal4” (VT004193.Gal4@attP2; ID 202884; VDRC), *w; Tk*-Gal4 (from J.A. Veenstra, Talence, France; Song et al., 2014), *yw; Sco/Cyo;* UAS-*dimm-myc* (from P.H. Taghert, St Louis, MO, Hewes et al., 2003), *w;* UAS-*dInR;* UAS-*dimm-myc* (a recombination made in this lab), *w;* UAS-*dilp6* (H. Stocker, Zürich, Switzerland; Ikeya et al., 2002), *w;* UAS-*dilp6*-RNAi [from The Vienna *Drosophila* Resource Center (VDRC), Vienna, Austria], *yw;* UAS-*mcd8-gfp* (from BDSC). Flies were kept under normal rearing conditions with 12:12 Light:Dark cycle at 25°C and fed a standard yeast, corn meal, agar containing food medium according to BDSC.

### Antisera and immunocytochemistry

The central nervous systems of third instar larvae and adult flies were fixed in ice-cold 4% paraformaldehyde in 0.1 M sodium phosphate buffer (PB; pH 7.4) for 2–4 h. After washing in PB 3 × 15 min the nervous tissues were dissected in PB. Tissues were then incubated in primary antibodies for 24–48 h at 4°C with gentle agitation. After washes (4 × 15 min) in PBS-Tx, secondary antibodies were applied for overnight or 48 h at 4°C. Tissues were washed in PBS-Tx 7 × 10 min, rinsed in 0.01 M PBS and mounted with 80% glycerol in 0.01 M PBS. The following primary antisera were used: rabbit anti-leucokinin (1:2000) (Cantera and Nässel, 1992; Nässel et al., 1992), rabbit-anti corazonin (1:4000; from J. A. Veenstra, Bordeaux, France; Veenstra and Davis, 1993), rabbit anti-pigment-dispersing hormone (1:3000; from H. Dircksen, Stockholm, Sweden; Dircksen et al., 1987), rabbit anti-crustacean cardioactive peptide (1:2000; from H. Dircksen; Dircksen et al., 1991), rabbit anti-eclosion hormone (1:500; from P. Taghert; Copenhaver and Truman, 1986), anti-PTTH (1:500; from P. Leopold; Nice, France; Yamanaka et al., 2013) rabbit anti-ion transport peptide (1:10,000; Dircksen et al., 2008), mouse anti-serotonin (1:80) (Clone 5HT-H209; Dako, Copenhagen, Denmark), mouse anti-chaoptin (1:2000; Developmental Studies Hybridoma Bank, University of Iowa; DSHB), mouse monoclonal anti-Bruchpilot (nc82; 1:20; DSHB; Schirmeier et al., 2016), guinea pig anti-DIMMED (1:2000; from P. Taghert, St Louis, Mo; Allan et al., 2005), rabbit anti-PHM (1:2000; from P. Taghert; Hewes et al., 2003; Park et al., 2008a), rabbit anti CAT-4 (1:2000; from P. Taghert; Park and Taghert, 2009), rabbit anti-LemTRP1 (Tachykinin; TK; 1:2000; Winther et al., 2003), mouse anti-tyrosine hydroxylase (1:200; Incstar Corp., Stillwater, MO; Nässel and Elekes, 1992), rabbit and mouse anti-GFP (1:1000) (Invitrogen, Carlsbad, CA). The following secondary antibodies (1:1000) were used: goat anti-rabbit Alexa 546, goat anti-rabbit Alexa 488, goat anti-mouse Alexa 488, goat anti-mouse Alexa 546 (all from Invitrogen), Cy5-tagged goat anti-rabbit antiserum, and Cy3-tagged goat anti-guinea pig antiserum (Jackson ImmunoResearch, West Grove, PA).

### Image analysis

Specimens were imaged with a Zeiss LSM 780 confocal microscope (Jena, Germany) using 20 ×, 40 × oil, or 63 × oil immersion objectives. Confocal images were processed with Zeiss LSM software for either projection of z-stacks or single optical sections. Images were in some cases edited for contrast and brightness in Adobe Photoshop CS3 Extended version 10.0. For cell size determination, the outline of each cell body was extracted and its area determined using Image J (freely available at http://rsb.info.nih.gov/ij/). For each genotype neurons in 5–15 animals from three independent crosses were measured.

### Locomotor activity recording

The *Drosophila* Activity Monitor System manufactured by TriKinetics Inc. USA was used to record locomotor activity and sleep duration. Flies were placed individually in glass tubes plugged with agar/sucrose medium (5% sucrose, 2% agar) as food at one end and sealed with paraffin wax, and at the other end the tubes were filled with small pieces of sponge. For each set of experiments, 32 glass tubes were inserted into a holder with infrared detectors. The software DAMsystem308 from TriKinetics Inc. was used for recording the locomotor activity (monitored as the number of infrared beam crossings per minute). Activity of each genotype was recorded for 5 days at 12L:12D and 8 days at D:D. A sleep-like resting state is defined here as continuous inactivity for at least 10 min. The interval time for collecting the sleep amount was 60 min. Average locomotor activity, total sleep amount and average sleep bout duration were calculated by using an Excel macros file.

### Statistical analysis

All statistical analyses were performed using Prism GraphPad 5.0. First all the data was checked with Shapiro-Wilk normality test and then analyzed with unpaired Students' *t*-test or ANOVA with Dunnett's multiple comparisons test if data was normally distributed. Non-parametric tests, Mann Whitney or Kruskal–Wallis test were performed when data did not show normal distribution.

## Results

### Expression of Dimm and dInR in Dimm-negative neurons in larvae affects the size of the cell body in a neuron type-specific manner

It was shown in previous study (Luo et al., 2013) that targeted manipulation of dInR expression affects cell size specifically in Dimm positive neurons, whereas it does not change the size of Dimm negative neurons. This was measured primarily as the size of the neuronal cell body, but it was also shown that axon terminations were affected. Note that the previous study showed that this induced growth is cell autonomous and independent of the size of surrounding neurons (Luo et al., 2013). Thus, we could induce a targeted size scaling among neurons that grow substantially with the increased size of the CNS during larval growth and development.

To further investigate the role of Dimm in size regulation, we expressed Dimm or Dimm with dInR in Dimm-negative neurons and analyzed cell body size in third instar larvae. Previously three groups of Dimm negative neurons: PTTH (prothoracicotropic hormone) producing neurons, 5-HT (serotonin) neurons, and motor neurons defined by the *OK6*-Gal4, were shown to display no changes in cell body size after dInR overexpression alone (Luo et al., 2013). Here we monitored cell body size in these neurons in third instar larvae after expression of Dimm alone or coexpression of Dimm and dInR. In the following we for simplicity use the term ectopic Dimm expression for directing UAS-*Dimm* to both Dimm-negative and Dimm positive neurons.

**Figure 1:**
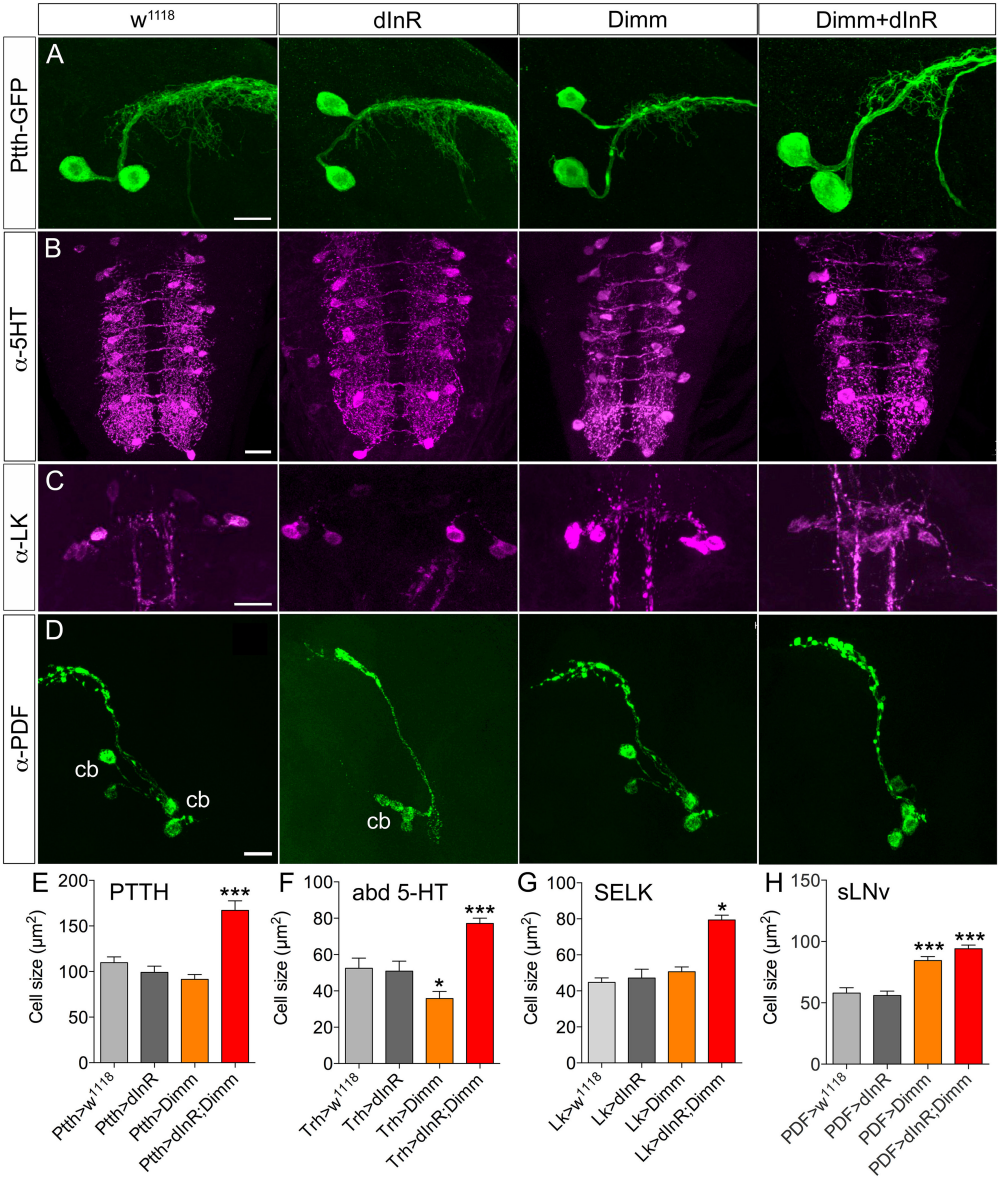
**Growth effects of ectopic expression of Dimm and dInR in Dimm-negative neurons in the third instar stage larva depend on neuron type. (A)** The Ptth-Gal4 driven GFP displays two PTTH neurons in each hemisphere of the larval brain. Expression dInR or Dimm alone has no effect on cell body size of PTTH neurons, whereas expression of Dimm/dInR triggers growth. **(B)** A Trh-Gal4 is used to drive Dimm and/or dInR expression in serotonin producing neurons. The cell body size of serotonin-immunoreactive neurons in the ventral nerve cord (VNC; abd 5-HT) decreases after Dimm expression, but increases after Dimm/dInR. **(C)** Leucokinin-immunoreactive neurons (SELKs) in the subesophageal ganglion are Dimm negative and display significant increase in cell size only after ectopic Dimm/dInR expression (using Lk-Gal4). **(D)** PDF-Gal4 is utilized for driving expression of Dimm and dInR in PDF-producing clock neurons (sLN_v_) of the brain. These neurons are located in ventral lateral part of the larval brain and have axon terminations dorsally. Both Dimm and Dimm/dInR expression give rise to larger cell bodies (cb) of sLNv neurons. **(E–H)** Quantification of cell body sizes after ectopic expression of Dimm, dInR or Dimm/dInR compared to controls (Gal4 crossed to *w*^*1118*^). Data are presented as means ± S.E.M, *n* = 6–36 flies for each genotype from three independent crosses (^*^p < 0.05, ^***^p < 0.001 as assessed by unpaired Students' *t*-test). Scale bar = 20 μm in **(A–D)**.

There are two large lateral neuroendocrine cells producing PTTH in each brain lobe that send axons toward the center of the brain and supply axon terminations to the ring gland (McBrayer et al., 2007). The cell body size did not change after ectopic Dimm expression in these PTTH neurons. However, these cell bodies became significantly larger after Dimm and dInR coexpression (Figures 1A,E). The thickness of the axons of these neurons also increased (Figure 1A). In Supplementary Figure 1 we show the localization of the PTTH neurons and all the different neuron types that were investigated in third instar larvae.

A group of 30 segmentally distributed serotonin-producing interneurons in abdominal neuromeres A1-A9 (Supplementary Figure 1), defined by a tryptophan hydroxylase (*Trh*) Gal4 driver (Alekseyenko et al., 2010), was next selected for analysis. Ectopic expression of Dimm with the *Trh*-Gal4 led to a slight decrease of cell body size, whereas combined Dimm and dInR expression increased the size of the cell bodies of A1-A9 neurons drastically (Figures 1B,F).

A set of segmental motor neurons (aCC neurons) in the larval ventral nerve cord (Supplementary Figure 1) defined by the RRa-Gal4 was analyzed next. Consistent with the lack of effect of expressing dInR in a broader set of motor neurons defined by the *OK6*-Gal4 (Luo et al., 2013), dInR expression alone did not affect the cell body size of aCC neurons in A1-A7 (Supplementary Figure 2). However, expression of Dimm alone caused a significant increase of cell body size. A further increase of cell body size was seen after combined Dimm and dInR expression (Supplementary Figure 2). It can be noted that, consistent with findings on histaminergic photoreceptors (Hamanaka et al., 2010), ectopic Dimm suppressed the expression of the endogenous neurotransmitter, which in aCCs is glutamate (unpublished findings).

Finally, we analyzed two other groups of Dimm negative peptidergic neurons in the brain: three pairs of subesophageal SELK neurons and four pairs of sLN_v_ (small ventral lateral clock neurons) in the brain. The SELK neurons express LK and the four sLN_v_ neurons produce pigment dispersing factor, PDF (Cantera and Nässel, 1992; Park et al., 2008b; de Haro et al., 2010). We used *Lk*-Gal4 and *Pdf* -Gal4 drivers to express Dimm and dInR (Figures 1C,D). The cell body size of SELK and sLNv neurons was not influenced by dInR alone, but increased after co-expression of Dimm and dInR (Figures 1G,H). Remarkably, expressing Dimm alone in sLN_v_ neurons resulted in larger cell bodies, but had no effect in SELK neurons (Figures 1G,H).

In summary, our data indicate that the cell body size of Dimm negative neurons in larvae is not affected by dInR expression alone, but consistently increases after ectopic co-expression of Dimm and dInR. Interestingly, expression of Dimm alone results in different size phenotypes depending on neuron type (see Table 1).

**Table 1:** **Cell body size of neurons in 3rd instar larvae**.

**Neuron type**		**Gal4**	**dInR**	**Dimm**	**Dimm + dInR**
DIMM+	ABLK	Lk	+^a^	NC	NC
	Abd CRZ	Crz	+	−	+
	vPDF	Pdf	+	NC	+
	LHLK	Lk	NC	NC	+
	CCAP	Ccap	NC^b^	+	NC
	EH	Eh	+	NC	+
	ITP (ipc-1)	Itp	NC	NC	+
DIMM−	SELK	Lk	NC	NC	+
	sLNv	Pdf	NC	+	++
	PTTH	Ptth	NC	NC	+
	5-HT	Trh	NC	−	+
	Motor neuron (aCC)	RRa	NC	+	++

a *Significant differences based on comparison to Gal4 > w^1118^. NC, no change; +, increase; ++, further increase; −, decrease*.

b *Data from Gu et al. (2014)*.

### Expression of Dimm and dInR in Dimm-positive neurons in larvae affects the size of their cell bodies depending on neuron type

We next screened neuron growth in a range of Dimm positive neurons in the third instar larva after targeted Dimm or Dimm and dInR expression.

**Figure 2:**
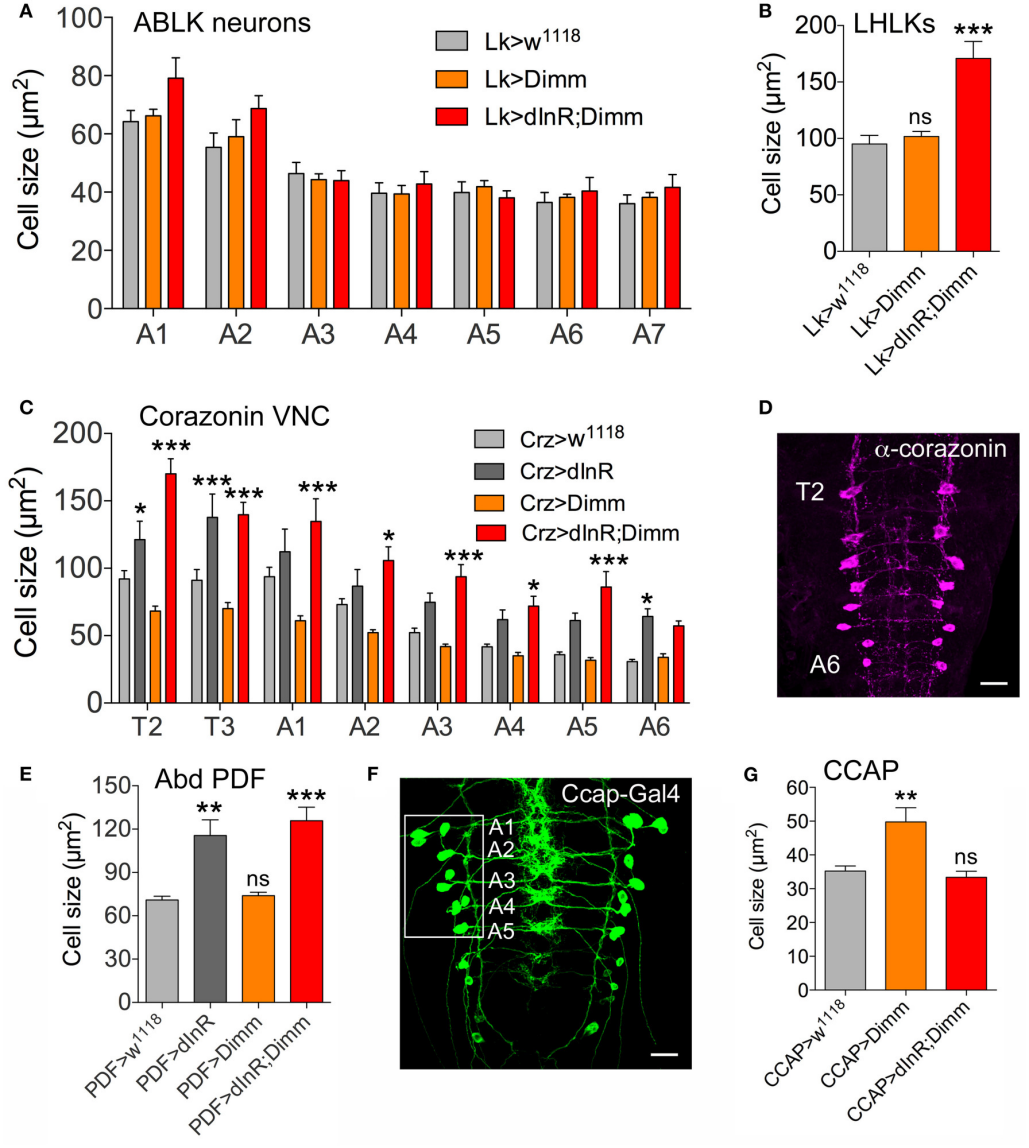
**Differential effects on cell body size of ectopic expression of Dimm, dInR, and Dimm/dInR in Dimm positive neurons in the third instar larva. (A)** ABLKs were analyzed in abdominal segments A1-A7 and no significant size change was detected. **(B)** Leucokinin neurons in the lateral horn (LHLKs) increased only after combined Dimm/dInR expression. **(C,D)** Corazonin-producing neurons (vCrz) in thoracic and abdominal segments (T2-A6) (shown in **D** after corazonin immunolabeling) displayed different growth phenotypes depending on segment. In most segments Dimm/dInR expression induced growth, whereas in some segments dInR alone also has this effect. **(E)** Cell bodies of PDF expressing neurons of abdominal ganglia (Abd PDF) increase in size after dInR and combined Dimm/dInR expression. **(F,G)** CCAP producing neurons in abdominal segments A-A5 (Ccap-Gal>GFP in **F**) increase in size only after Dimm expression **(G)**. Data are presented as means ± S.E.M, *n* = 6–13 flies for each genotype from three independent crosses (^*^p < 0.05, ^**^p < 0.01, ^***^p < 0.001 as assessed by unpaired Students' *t*-test or non-parametric Mann Whitney test when data was not normally distributed). Further images of neurons from these experiments are shown in Supplementary Figure 5.

Analysis of the 14 abdominal leucokinin (LK) producing cells (ABLKs), in A1-A7 of the ventral nerve cord, and one pair of LK cells in the lateral horn of the brain (LHLKs) after expression of Dimm alone show no effect on cell body size (Figures 2A,B, Supplementary Figures 3A,B). However, combined Dimm and dInR expression induced growth in LHLKs (Figure 2B, Supplementary Figure 3B), whereas no change could be seen in ABLK neurons (Figure 2A, Supplementary Figure 3A). Earlier we had shown that ectopic expression of dInR alone leads to a 65% increase in size of ABLK cell bodies (Luo et al., 2013) (see also Supplementary Figure 3A).

A set of 16 ventral corazonin producing neurons (vCrz) in larval thoracic (T2, T3) and abdominal neuromeres (A1-A6) also expresses Dimm (Park et al., 2008a). Ectopic dInR expression led to enlarged size of vCrz neurons (Figures 2C,D, Supplementary Figure 3C). However, Dimm expression alone caused a decrease in cell body size, whereas Dimm combined with dInR resulted in an increase (Figures 2C,D, Supplementary Figure 3C).

Two other groups of Dimm positive cells in the larva, a set of six abdominal PDF cells (vPDF) (Figure 2E, Supplementary Figure 3D) and one pair of ventromedial neurosecretory cells producing eclosion hormone (EH) (Supplementary Figure 4) responded similarly to targeted expression. Both types displayed no alteration in cell body size with Dimm alone, but a significant increase after dInR as well as Dimm/dInR expression (Figure 2F, Supplementary Figures 3D, 4B,D).

Next we monitored segmental neurons producing CCAP in the ventral nerve cord. To determine which of the CCAP neurons that expresses Dimm, we drove *mCD8*-GFP under the control of *Ccap*-Gal4 and labeled with anti-CCAP, anti-Dimm, and anti-GFP (Figure 2F, Supplementary Figure 5). In each hemi segment of the abdominal neuromeres A1-A7, seven interneurons and seven efferent neurons produce CCAP. We found that efferent CCAP neurons express Dimm prominently only in A1-A5, whereas Dimm expression was very weak in CCAP interneurons (Supplementary Figure 5A). We monitored cell body size of efferent CCAP neurons in A1-A5. Dimm alone induced growth, but Dimm with dInR did not affect cell body size (Figure 2G, Supplementary Figure 5B).

**Figure 3:**
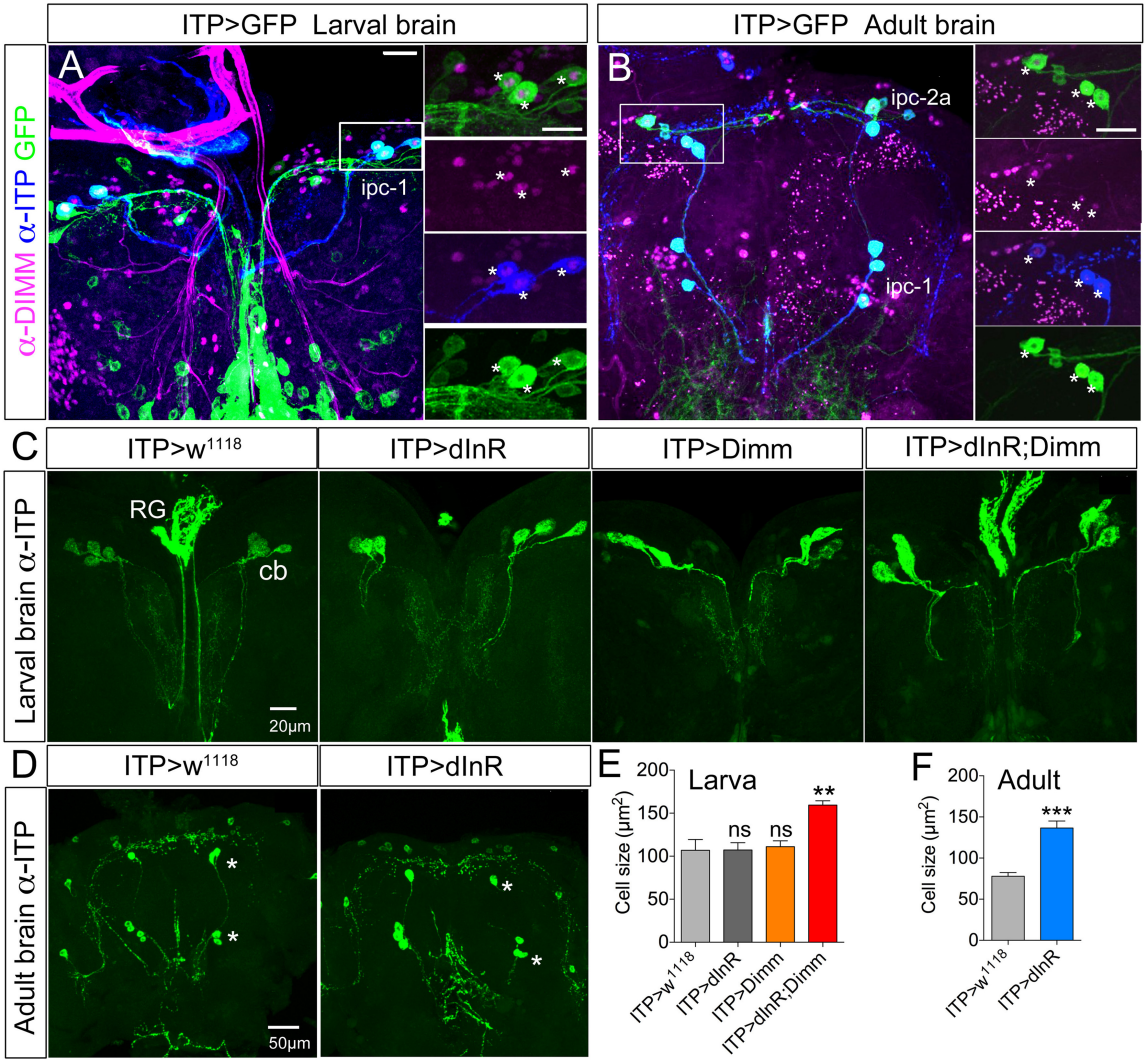
**In both larvae and adults ion transport peptide (ITP) expressing neurons are Dimm positive, and ectopic Dimm/dInR triggers growth**. **(A)** In the larval dorsal brain three pairs of ITP positive neurons (ipc-1; stained with anti-ITP, blue) also display GFP expression under the control of ITP-Gal4. Enlarged panels on the right show details. Dimm is expressed in ITP positive neurons (asterisks). **(B)** In the adult brain ITP positive neurons (ipc-1 and ipc-2a) are seen with both GFP and ITP immunostaining. Ipc-1 as well as ipc-2a neurons display Dimm expression in the nucleus (right panels). **(C)** Only ectopic Dimm/dInR expression leads to significant growth of larval ipc-1 neurons. RG, ring gland; cb, cell body. **(D)** In the adult brain the ITP positive neurons display enlarged cell bodies after dInR expression. However, after both Dimm and Dimm/dInR expression the flies die before adult eclosion. **(E)** Cell body sizes after Dimm, dInR, or Dimm/dInR ectopic expression in larvae compared to controls. Data are presented as means ± S.E.M, *n* = 6–7 flies for each genotype from three independent crosses (^**^p < 0.01; ns, not significant as assessed by unpaired Students' *t*-test). **(F)** Cell body sizes after dInR expression compared to controls in adult flies. Data are presented as means ± S.E.M, *n* = 10 flies for each genotype from three independent crosses (^***^p < 0.001 as assessed by unpaired Students' *t*-test). Scale bar = 20 μm in **(A–C)**, 50 μm in **(D)**.

Ion transport peptide (ITP) expressing neurons have been shown to be Dimm positive in a previous study (Park et al., 2008b). Here we utilized a Gal4 driver including brain ITP neurons (we refer to this as *ITP*-Gal4; see Section Materials and Methods) and anti ITP antibody for analysis. Dimm is expressed in ITP positive neurons (ipc-1) in larvae as well as in ipc-1 and ipc-2a neurons in the adult (Figures 3A,B). Increased size of larval ipc-1 neurons was only observed after Dimm/dInR expression, whereas no effect was seen with expression of Dimm or dInR alone (Figures 3C,E). Surprisingly, ITP-Gal4-driven Dimm and Dimm/dInR expression caused lethality in pupae, whereas with dInR alone flies developed into adults. This could be due to the strong but additional ectopic *ITP*-Gal4 expression in neurons of the ventral nerve cord, which does not match the ITP immunolabeling (not shown). Thus, Dimm and Dimm/dInR expression might affect functions of those sets of neurons and lead to developmental failure. We could, however, investigate the effect of dInR expression in adult ITP neurons: they displayed larger cell bodies compared to controls (Figures 3D,F). Two other Gal4 drivers that include ipc-1 neurons were also employed for analysis of effects of dInR expression in larvae: *c929*-Gal4 and *Kurs6*. We obtained similar results on ITP neuron growth as with the *ITP*-Gal4 (not shown). As summarized in Table 1, we find that the cell body size of Dimm positive neurons, in most cases increase with expression of dInR, and Dimm/dInR.

### Targeted expression of Dimm and Dimm/dInR in Dimm-negative neurons affects cell size also in adults

**Figure 4:**
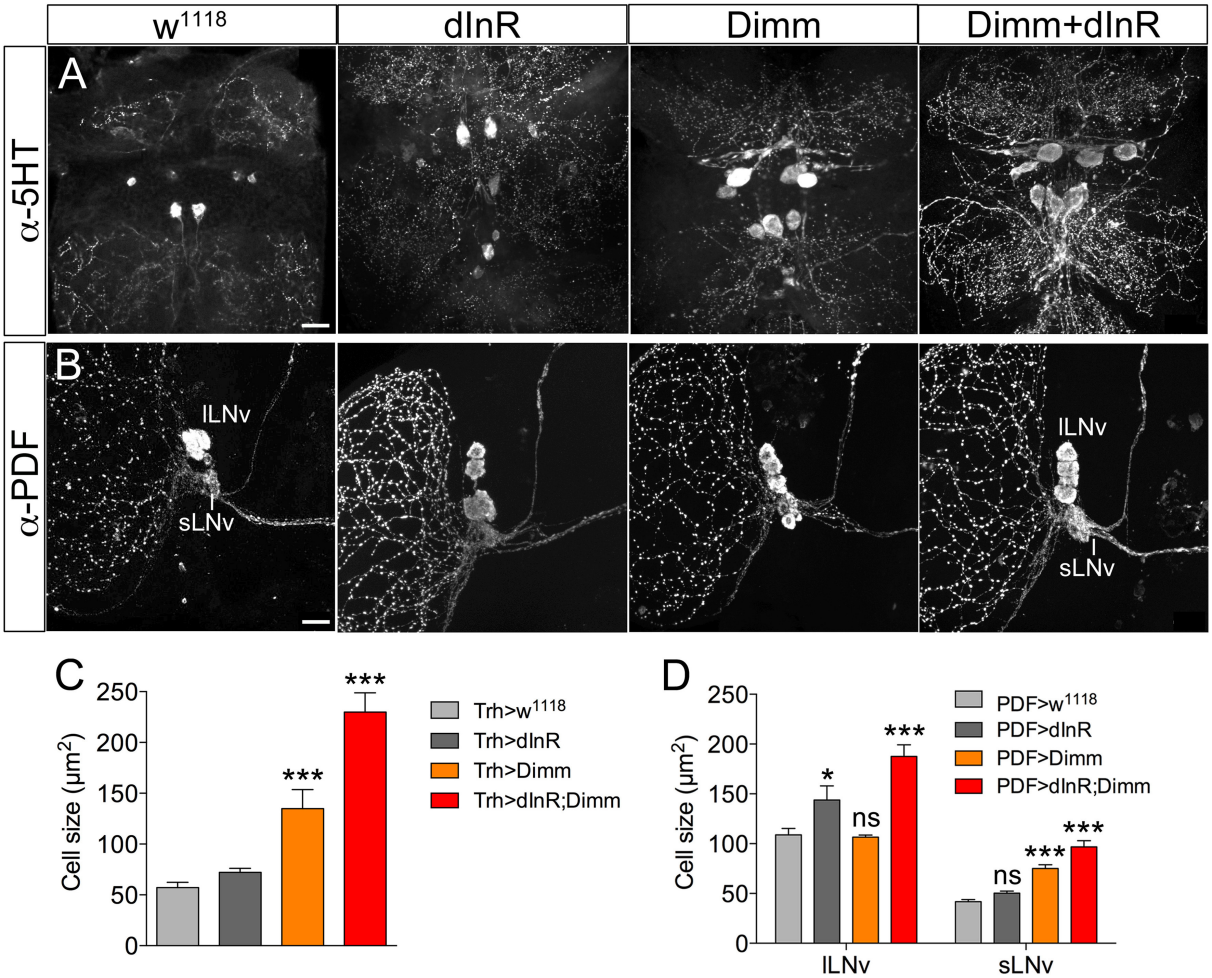
**Ectopic expression of Dimm and Dimm/dInR in Dimm negative neurons affects cell size also in 3-day-old adults**. **(A)** Eight serotonin-immunoreactive neurons located between thoracic segments T1 and T2 in the VNC obtain larger cell bodies after expression of Dimm alone and further increase in size after Dimm/dInR expression (using a Trh-Gal4 driver). **(B)** The PDF immunoreactive clock neurons, small ventral lateral neurons (sLN_v_) are Dimm negative, whereas large ventral lateral neurons (lLN_v_) are Dimm positive. Expression of Dimm alone (with a Pdf-Gal4) causes an increase of cell body size in sLN_v_s, but not in lLN_v_s, and Dimm/dInR further promoted cell growth in both cell groups. **(C,D)** Cell body sizes of serotonergic and clock neurons after ectopic expression of dInR, Dimm, or Dimm/dInR compared to controls. Data are presented as means ± S.E.M, *n* = 6–13 flies for each genotype from three independent crosses (^*^p < 0.05, ^***^p < 0.001; ns, not significant as assessed by unpaired Students' *t*-test or non-parametric Mann Whitney test when data was not normally distributed). Scale bar = 20 μm in **(A,B)**.

We next asked whether also adult neurons are affected by expression of Dimm and dInR and analyzed cell body size in both Dimm negative and positive neurons in 3-day-old flies. In Supplementary Figure 6 a schematic diagram of the different adult neuron types investigated are shown. Three groups of Dimm negative neurons tested, SELK, sLN_*v*_s, and serotonin expressing neurons in thoracic segment T2, responded similarly to Dimm and Dimm/dInR overexpression. Their cell bodies increased in size after Dimm expression and increased further with Dimm/dInR (Figure 4).

To further explore different types of Dimm negative neurons we expressed Dimm in olfactory sensory neurons (OSNs) innervating the antennal lobe, photoreceptors of the compound eye, as well as enteroendocrine (EE) cells in the midgut. To analyze growth of OSN axon terminations we employed the widely expressed *Orco*-Gal4 (Larsson et al., 2004) to drive dInR, or Dimm and dInR, and monitored the size of the entire antennal lobe, as well as a single glomerulus, the dorsal lateral glomerulus 3 (DL3) that is easy to delineate with the markers employed. The antennal lobe and DL3 glomerulus were stained by anti Bruchpilot and sNPF antibody (see Carlsson et al., 2010; Supplementary Figures 7A,B). Neither the size of the antennal lobe nor the DL3 glomerulus was affected by dInR alone, whereas Dimm alone increased the size of both structures and so did Dimm with dInR (Table 2, Supplementary Figures 7B–E). The global growth of the antennal lobe is likely to be the result of the size increase of a subset of the total glomeruli since the Orco-Gal4 is not expressed in all of these (Larsson et al., 2004).

**Table 2:** **Cell body size of neurons in adult flies**.

**Neuron/neuropil type**		**Gal4**	**dInR**	**Dimm**	**Dimm+ dInR**
Dimm positive	ABLK	Lk	+^a^	+	++
	Conditional ABLK	Lk +Gal80	+	NC	+
	DLP (CRZ)	Crz	+	+	++
	l-LNv	Pdf	+	NC	+
	vPDF	Pdf	+	+	++
	LHLK	Lk	NC	NC	+
	Conditional LHLK	Lk +Gal80	NC	NC	NC
	CCAP (brain)	Ccap	+	+	++
	vCCAP	Ccap	+	+	++
	ITP	Itp	+	Lethal	Lethal
	Abd CRZ (female)^b^	Crz	Missing	Present	+
	EH^b^	Eh	Missing	Present	+
	CCAP (SEG)^b^	Ccap	Missing	Present	+
Dimm negative	SELK	Lk	NC	+	++
	Conditional SELK	Lk +Gal80	NC	NC	+
	PTTH^b^	Ptth	Missing	Present	+
	s-LNv	Pdf	NC	+	++
	5HT Thoracic 2	Trh	NC	+	++
	R7/8 (pupa)	GMR	+	Lethal	Lethal
	EE cells (midgut)	Tk	+	NC	++
Neuropil	Antennal lobe	Orco	NC	+	+
	DL3 glomerulus	Orco	NC	+	+

a *Significant differences based on comparison to Gal4 > w^1118^. NC, no change; +, increase; ++, further increase*.

b *These neurons normally undergo programmed cell death during metamorphosis or first day(s) of adult stage (missing). Ectopic Dimm expression leads to block of apoptosis (present)*.

For manipulations of photoreceptor cells we utilized the *glass* reporter, *longGMR*-Gal4 as a driver (Moses and Rubin, 1991; Hamanaka et al., 2010), and visualized photoreceptor axons with antiserum to Chaoptin, a photoreceptor cell-specific membrane glycoprotein. There are eight photoreceptor cells (R1-R8) in each ommatidium, which extend axons from the retina into the optic lobe. R1-R6 axons terminate in the lamina, while R7 and R8 send their axons further into medulla (see Meinertzhagen and Hanson, 1993; Fischbach and Hiesinger, 2008). Here we monitored the size of axon terminations of R7/R8 in the medulla. In the third instar larvae of these flies the structure of eye disc was abnormal, and the characteristic morphogenetic furrow and well-organized patterning of ommatidial clusters were missing (Supplementary Figures 8A–D). Differentiated photoreceptors failed to form the hexagonal array in the eye disc (Supplementary Figures 8E–H). Some of these cells still send axons through the optic stalk into the developing lamina and medulla (Supplementary Figures 8C,D). Overexpression of Dimm with *LongGMR*-Gal4 leads to lethality due to failure to eclose as adults. We also utilized a recombinant line: *GMR*-Gal4; UAS-*Dimm*, and surprisingly this recombinant fly can develop into adulthood and is fertile. Thus, we looked into the pupal stage of these flies. In pupae the ectopically Dimm expressing axons of R7/R8 photoreceptors in the medulla are disrupted and do not form the ordinary pattern (Supplementary Figures 9A–C). Expressing the dInR alone in photoreceptors resulted in thicker axon terminations of R7/R8 in pupae and also a slightly irregular axon projection into the medulla (Supplementary Figures 9C,E). The compound eyes of these pupae did not develop properly and displayed fewer ommatidia than control flies (Supplementary Figure 9D).

**Figure 5:**
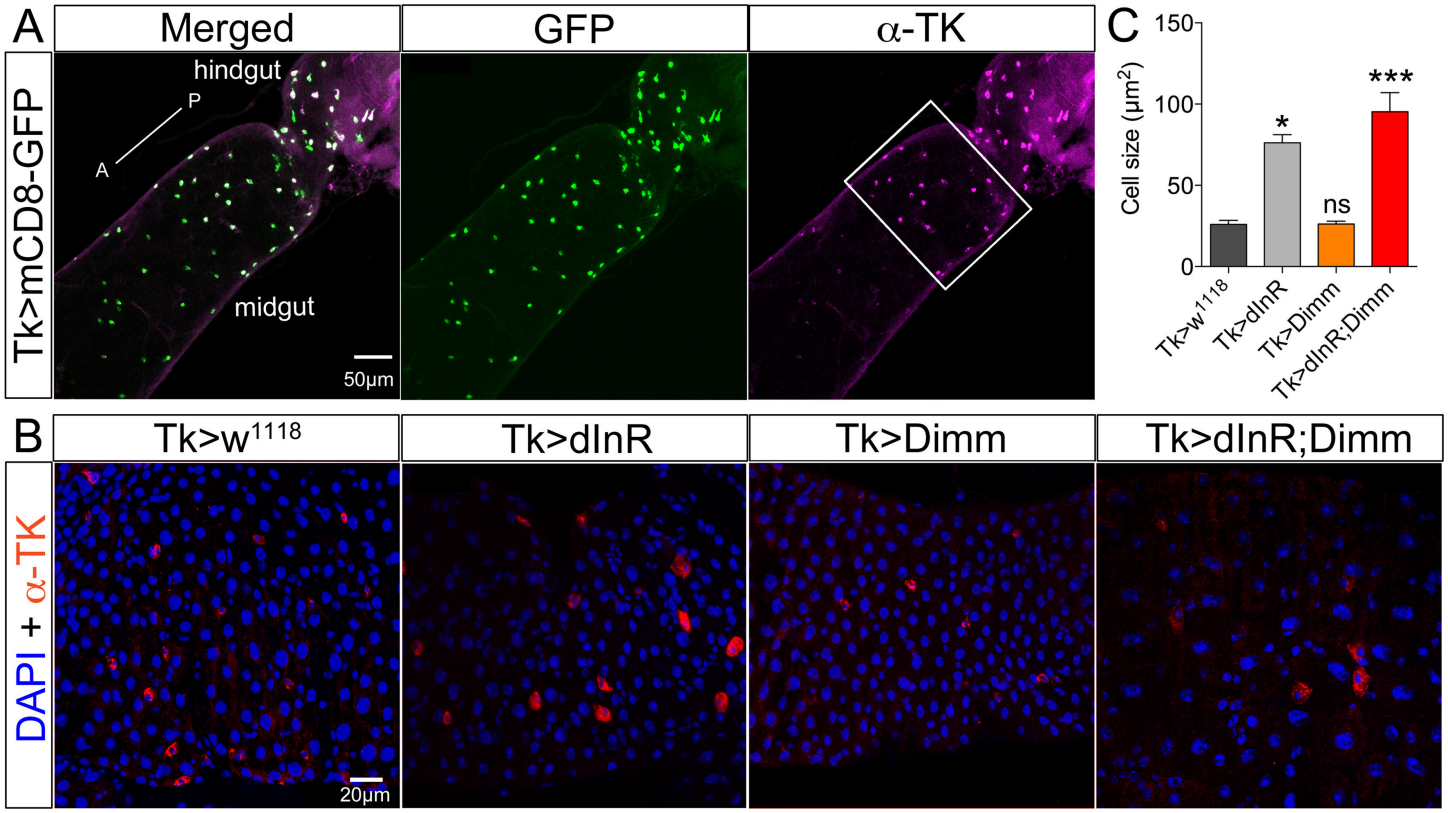
**Tachykinin (TK) expressing enteroendocrine (EE) cells in adult midgut are Dimm negative and grow after ectopic expression of dInR and Dimm/dInR**. **(A)** TK immunolabeling (magenta) in posterior midgut and anterior hindgut is colocalized in EEs with GFP expression driven by Tk-Gal4. **(B)** Posterior region of midgut (white bracket in **A**) was monitored after manipulations. Nuclei were marked with DAPI (blue) and EE cells were stained with anti-TK antibody (red). Cell sizes increased significantly after dInR expression and Dimm/dInR expression, compared to control, and Dimm alone. **(C)** Cell body sizes after dInR, Dimm, and Dimm/dInR expression compared to controls. Data are presented as means ± S.E.M, *n* = 6–9 flies for each genotype from three independent crosses (^*^p < 0.05, ^***^p < 0.001 as assessed by unpaired Students' *t*-test or non-parametric Mann Whitney test when data was not normally distributed). Scale bar = 50 μm in **(A)**, 20 μm in **(B)**.

Different populations of midgut enteroendocrine (EE) cells express various neuropeptides (Veenstra et al., 2008), such as for instance tachykinin (TK) (see Siviter et al., 2000; Song et al., 2014). Here we utilized a *Tk*-Gal4 (Song et al., 2014) to specifically target expression to subsets of midgut EE cells producing TK. *Tk*-Gal4 driven GFP was found colocalized with TK-immunolabeling in the posterior midgut as well as the anterior of hindgut in adult flies (Figure 5A). Both dInR alone and Dimm/dInR expression targeted to EE cells induced significant size increase of TK expressing gut cells, whereas Dimm alone had no effect (Figures 5B,C).

**Figure 6:**
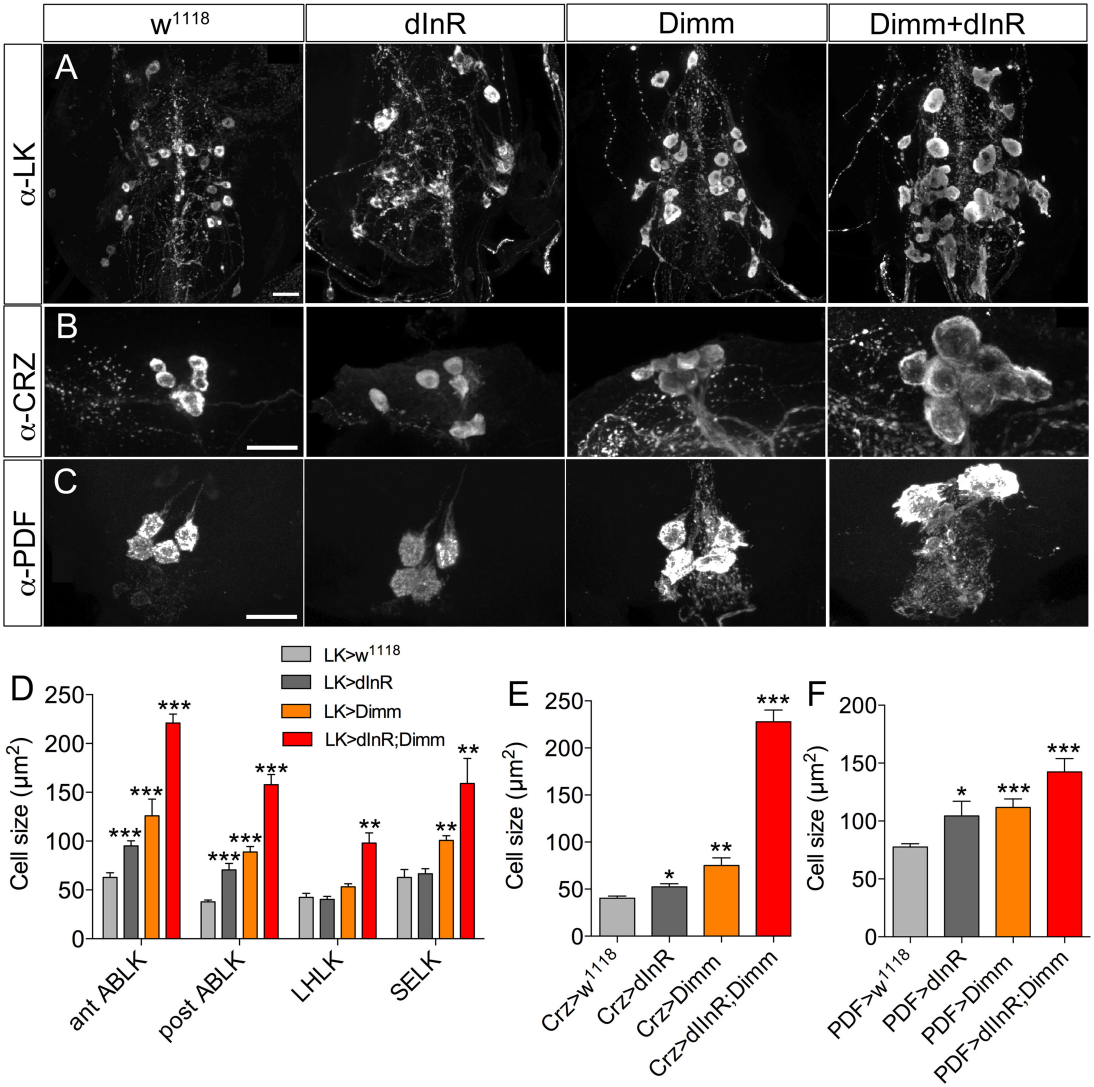
**Growth of Dimm positive neurons in 3-day-old adult flies after ectopic dInR, Dimm, and Dimm/dInR expression. (A)** Analysis of posterior ABLKs (post ABLK) and adult-specific anterior ABLKs (ant ABLK) reveals a size increase both after expression of dInR or Dimm alone, and further growth with Dimm/dInR (see Figure 6D for quantification). **(B)** Expression of dInR or Dimm alone causes growth in dorsal lateral posterior corazonin expressing neurons (DLP), and Dimm/dInR increases the size further (see also **E**). **(C)** Four PDF positive neurons posteriorly in the adult abdominal ganglia also grow in response to the three manipulations (see also **F**). **(D–F)** Cell sizes after dInR, Dimm, and Dimm/dInR expression compared with controls. In **(D)** also the leucokinin neurons LHLK and SELK are shown. Data are presented as means ± S.E.M, *n* = 6–11 flies for each genotype from three crosses (^*^p < 0.05, ^**^p < 0.01, ^***^p < 0.001 as assessed by unpaired Students' *t*-test or non-parametric Mann Whitney test when data was not normally distributed). Scale bar = 20 μm in **(A–C)**.

We also analyzed Dimm positive neurons in 3-day-old adult flies after manipulations of Dimm and dInR (Figure 6). The leucokinin-producing ABLK neurons (Figure 6A), dorsal lateral peptidergic neurons (DLP) expressing corazonin (Figure 6B), and pigment-dispersing factor expressing neurons (abd PDF) of abdominal ganglia (Figure 6C) all displayed an increase of cell body size after both ectopic dInR and Dimm expression, and Dimm/dInR promoted a further growth (Figures 4D, 6D).

Two groups of neurons in the adult brain responded differently to manipulations. The four large ventral lateral PDF-producing neurons (l-LN_v_s) (Figures 5B,C), and one pair of lateral horn leucokinin (LHLK) neurons in each hemisphere, were not affected by Dimm alone but displayed larger cell bodies after dInR and Dimm/dInR expression (**Figure 8D**). The above findings are summarized in Table 2.

**Figure 7:**
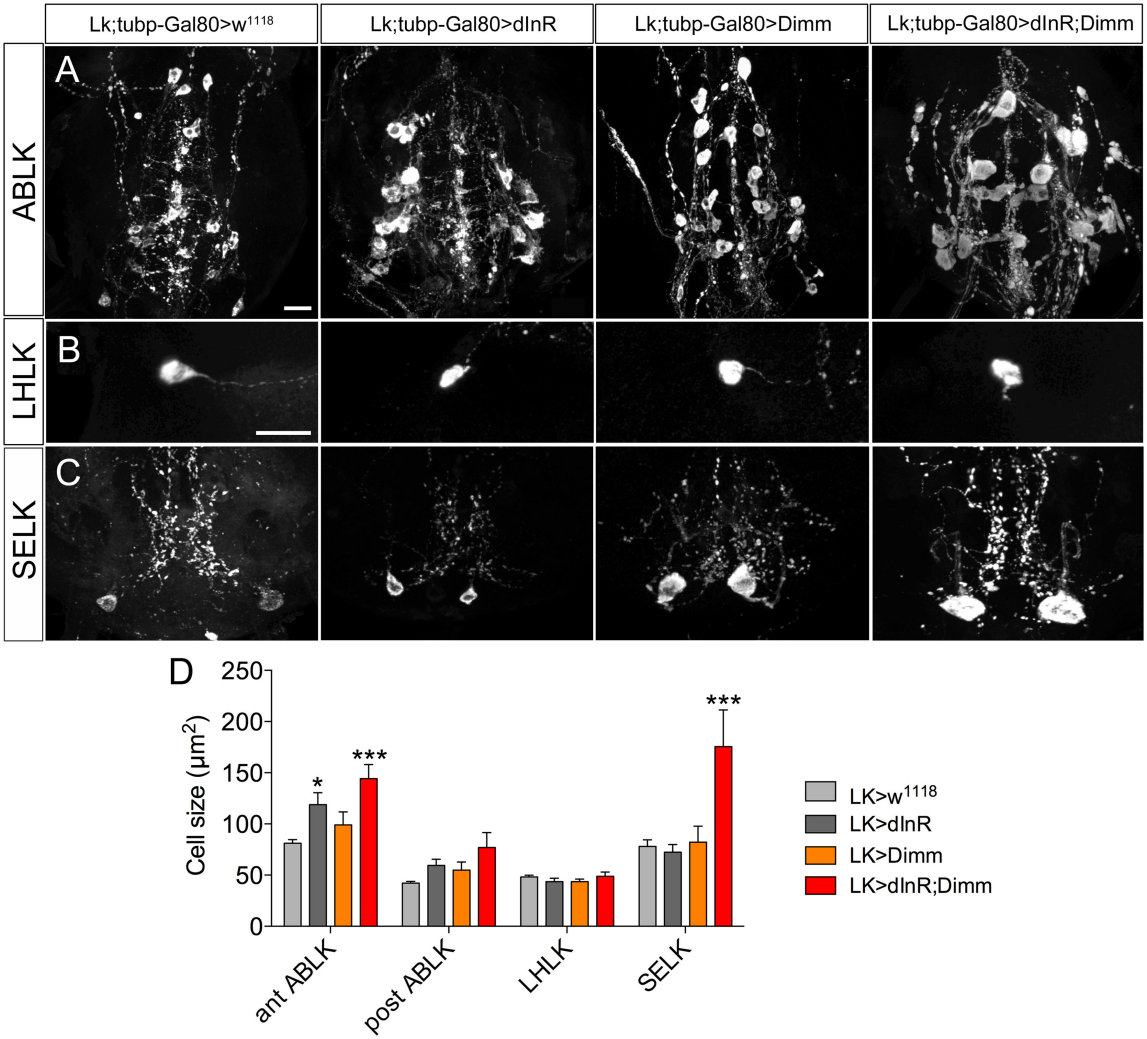
**Growth effects of conditional expression of dInR, Dimm, and Dimm/dInR in leucokinin positive neurons in adult flies**. To trigger Dimm expression in the adult the temperature inducible Gal80 system was used. One-day-old flies were transferred to 30°C for 10 days to activate Gal4 expression. Neuron sizes were monitored in 10-day-old flies. **(A)** Conditional dInR expression alone gives rise to a significant increase in cell body size in anterior ABLKs, and Dimm/dInR increases cell bodies further, whereas posterior ABLKs are not affected (see also **D**). **(B)** No significant difference is seen in cell body size of LHLKs after any manipulation. **(C)** SELKs display a significant size increase only after ectopic Dimm/dInR expression. **(D)** Quantification of cell-body sizes of the neurons in **(A–C)** after conditional dInR, Dimm, and Dimm/dInR expression. Data are presented as means ± S.E.M, *n* = 7–13 flies for each genotype from three crosses (^*^p < 0.05, ^***^p < 0.001 as assessed by unpaired Students' *t*-test). Scale bar = 20 μm in **(A–C)**.

To test whether targeted expression of Dimm and dInR specifically in the adult stage has an effect on neuron size, we utilized a temperature sensitive *Lk*-Gal4; *tubP*-Gal80 driver to conditionally target gene expression in adult flies. Crossed flies were raised at 20°C and 1-day-old adult flies were transferred to 30°C for 10 days after which the cell body sizes were measured (Figure 7). We observed larger cell bodies of both anterior and posterior ABLK neurons after adult-specific ectopic dInR expression (Figures 7A,D). However, there was no significant difference in cell body size of SELKs and LHLKs after conditional Dimm expression (Figures 7B–D). Only when Dimm was expressed together with dInR the cell body size of anterior ABLKs and SELKs increased significantly (Figure 7D). These results suggest that the dInR can regulate the cell body size of ABLKs in adult flies, but not in LHLKs or SELKs. Dimm expression alone cannot increase the cell body size of any of the LK expressing neurons. When co-expressing Dimm and dInR, the anterior ABLKs and SELKs grew drastically bigger, but not posterior ABLKs or LHLKs. Our data therefore suggest that Dimm action requires increased dInR expression to affect growth of SELKs in adults, whereas ectopic Dimm is not sufficient for LK neurons to trigger growth in adults. See Table 2 for summary.

### Ectopic Dimm expression blocks programmed apoptosis in a range of neurons

A range of neurons in the larval central nervous system undergoes programmed cell death (PCD) after puparium formation or after adult emergence (Hidalgo and ffrench-Constant, 2003; Hay et al., 2004; Choi et al., 2006). After puparium formation apoptosis takes place mainly in the ventral nerve cord. Corazonin-producing neurons (vCrz neurons) of the ventral nerve cord are among these neurons. They die within 6–7 h after puparium formation in female flies, and the process is caspase-dependent (Choi et al., 2006; Lee et al., 2011; Lee G. et al., 2013). Many eclosion-behavior associated peptidergic neurons are eliminated shortly after adult emergence (Robinow et al., 1993, 1997; Helfrich-Förster, 1997; Draizen et al., 1999; Renn et al., 1999). For instance CCAP producing neurons located in the subesophageal ganglion and another set of CCAP neurons in the ventral nerve cord undergo apoptosis 3–5 days after adult eclosion (Lee G. G. et al., 2013). Eclosion hormone (EH) is expressed in a pair of brain cells in the larva, and in the pharate adult until 8 h before ecdysis (Horodyski et al., 1993). Finally, four PTTH producing neurons are detectable in the brain until about 24 h after adult eclosion, but cannot be seen in 3-day-old adult flies or later.

**Figure 8:**
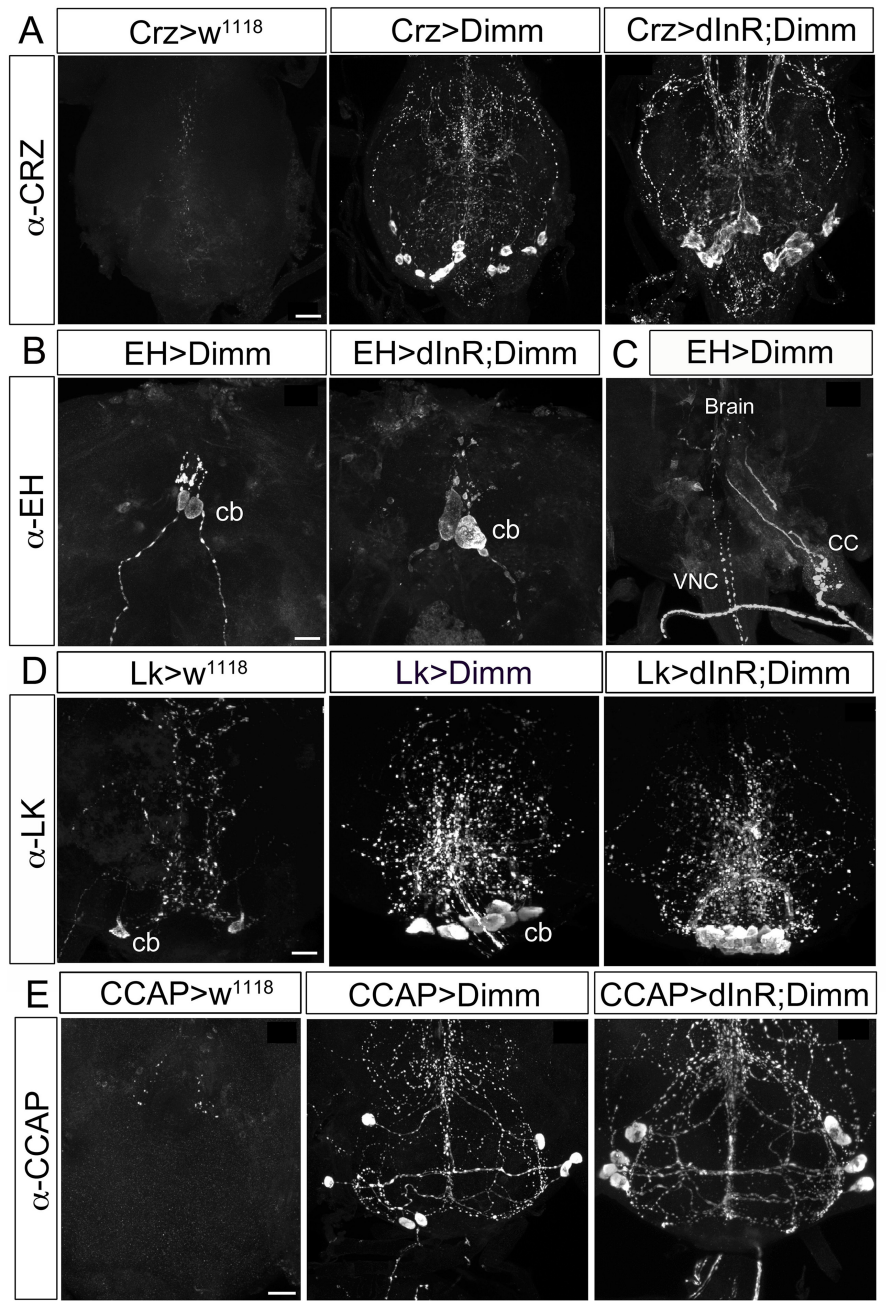
**Ectopic Dimm expression blocks programmed cell death in a range of neurons as monitored in adult flies. (A)** No corazonin immunoreactivity is detectable in the abdominal ganglia of control (Crz > *w*^*1118*^) female flies, but there are 14–16 corazonin positive neurons present in the T3 segment of VNC after Dimm expression using a Crz-Gal4 driver. These neurons become larger after expressing combined Dimm/dInR. **(B,C)** EH expressing neurons also bypass apoptosis after Dimm expression (EH-Gal4 driver) and the size of their cell body increases after Dimm/dInR expression (controls not shown). The axons extending to the ring gland in larvae could be observed in adults terminating in the corpora cardiaca (CC) whereas the other set projected to the VNC. **(D)** Only one pair of leucokinin-producing SELKs is present in control adult flies (Lk > *w*^*1118*^), whereas two or three pairs of SELKs are seen in the larval stage (not shown). After ectopic Dimm expression, three pairs of SELKs remain and become larger. Ectopic Dimm/dInR causes further increased cell body size. **(E)** The CCAP-immunoreactive neurons in the subesophageal ganglion normally undergo apoptosis within 3–5 days after adult emergence. Ectopic Dimm expression blocks this programmed cell death, and Dimm/dInR induces further cell growth of CCAP neurons. Scale bar = 20 μm.

**Figure 9:**
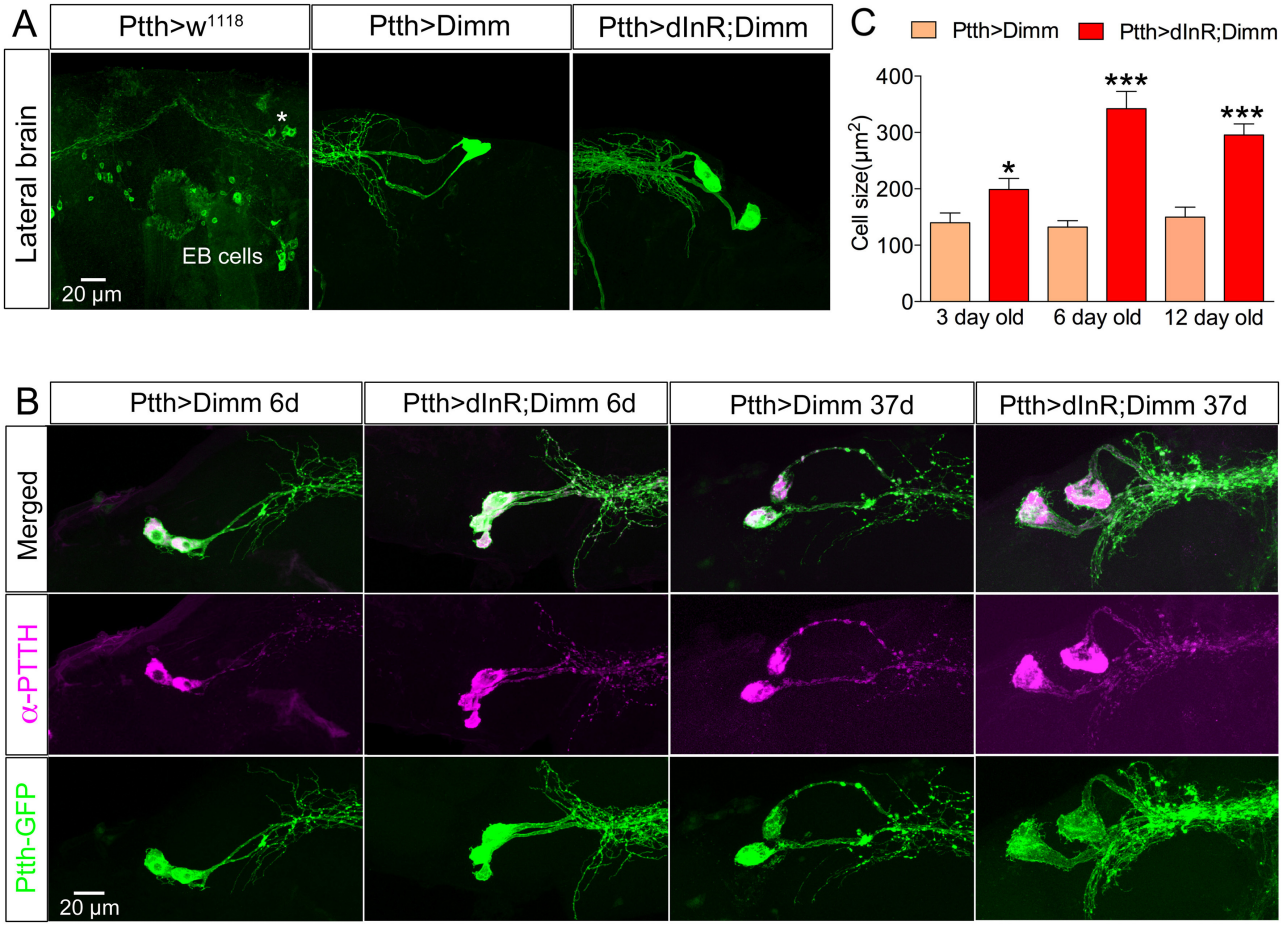
**Larval PTTH producing neurons survive long into adulthood after Dimm and Dimm/dInR expression. (A)** Larval PTTH positive neurons are not detectable 1 day after adult eclosion in controls (Ptth > *w*^*1118*^) or wild type flies. Instead, a set of R-neurons (EB cells) innervating the ellipsoid body express GFP (but are PTTH immuno-negative). After Dimm expression larval-derived PTTH neurons persist laterally in the adult brain of 3-day-old adult flies, and ectopic Dimm/dInR induces larger cell body size. **(B)** PTTH is still produced in the surviving larval neurons in 6 and 37 day old flies expressing Dimm and Dimm/dInR under control of the Ptth-Gal4 (anti-PTTH, magenta). The PTTH neurons continuously grow and in 37-day-old flies they display a cell body size much larger than in 3 and 6 day old flies. **(C)** Quantification of cell-body sizes after Dimm and Dimm/dInR expression in 3, 6, and 12 d old flies. Data are presented as means ± S.E.M, *n* = 6–11 flies for each genotype from three crosses (^*^p < 0.05, ^***^p < 0.001 as assessed by unpaired Students' *t*-test or non-parametric Mann Whitney test when data was not normally distributed). Scale bar = 20 μm in **(A,B)**.

**Figure 10:**
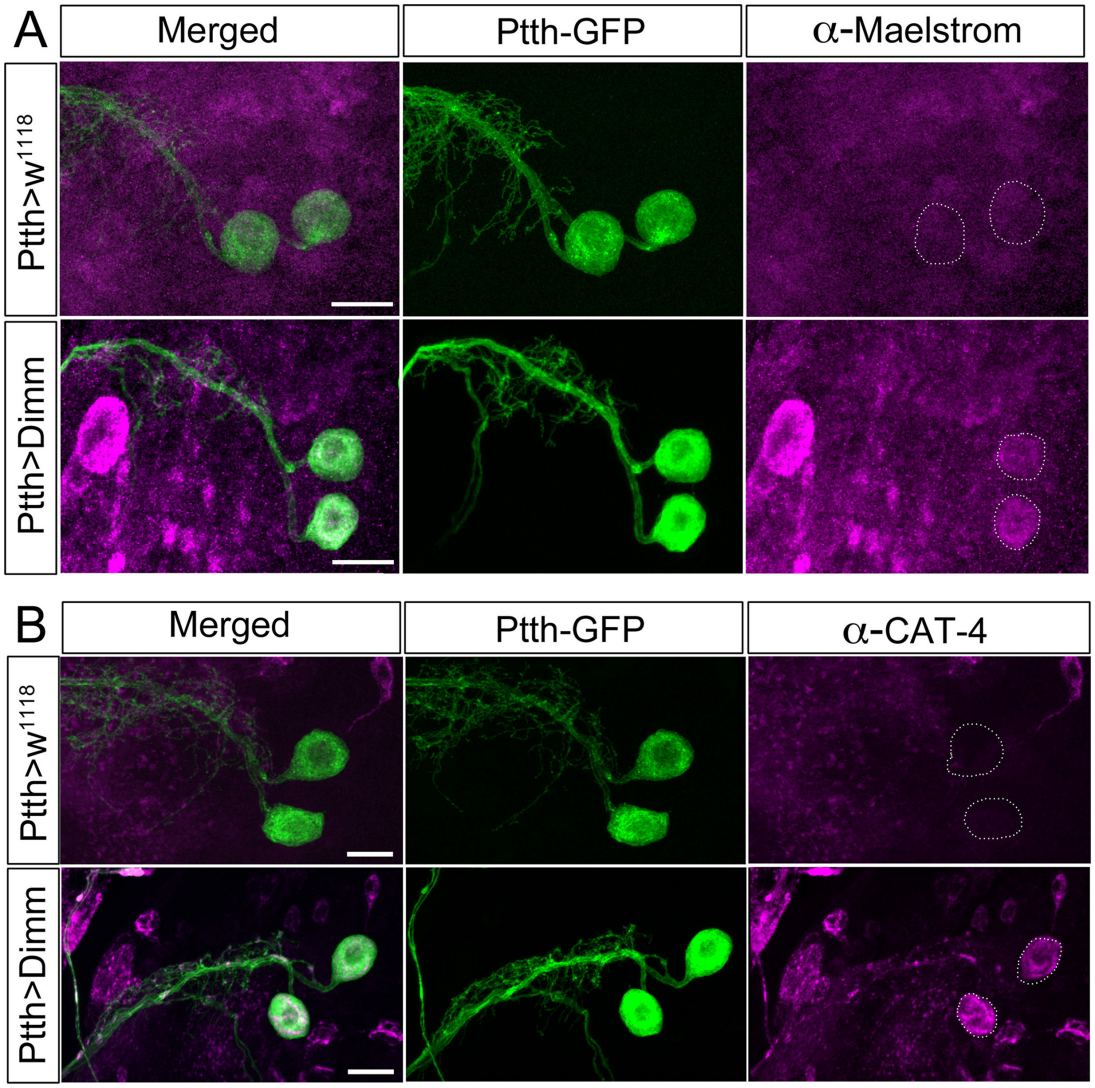
**The direct Dimm targets Maelstrom and CAT-4 are upregulated after ectopic expression of Dimm in PTTH producing neurons**. **(A)** Maelstrom (anti-Maelstrom, magenta) is not expressed in wild type PTTH neurons in larvae (GFP, green). After ectopic Dimm expression maelstrom immunofluorescence can be detected in the PTTH neurons (dashed circle). **(B)** Anti-CAT-4 (CAT-4, magenta) labels PTTH neurons after Dimm expression, whereas no detectable CAT-4 immunostaining is seen in controls (dashed circle). Scale bar = 20 μm.

Interestingly, we observed that these neurons, which should normally undergo apoptosis, are still present in aged adult flies after ectopic Dimm and Dimm/dInR expression (Figures 8, 9). In controls, no corazonin immunoreactivity was detectable in the ventral nerve cord of 3-day-old adult female flies. After Dimm expression, however, there were 14–16 corazonin positive neurons present in the T3 segment of the ventral nerve cord (Figure 10A). Furthermore, these neurons remained and became even larger after expressing Dimm/dInR (Figure 8A). Since the location and number of these cells are consistent with the larval corazonin producing neurons, our results indicate that these neurons survive into the adult stage due to ectopic Dimm.

The same observation was made after Dimm expression in CCAP and EH producing neurons, as well as the leucokinin producing SELKs (Figures 8B–D). Seven neurons expressing CCAP are seen in the subesophageal ganglion (SEG) 6 h after eclosion and later undergo PCD at 3–4 d after eclosion (Park et al., 2003). These neurons were still detectable in 3-day-old flies after Dimm expression whereas they could be seen neither in control flies nor after dInR expression (Figure 8D). The CCAP neurons in the ventral nerve cord (VNC) also bypassed apoptosis after Dimm expression (Supplementary Figures 10A–D). However, the *CCAP*-Gal4 line used has been shown to confer an anti-apoptotic effect on these CCAP neurons in ventral nerve cord (Park et al., 2003), but not the SEG neurons. Thus, there were still a few of the CCAP neurons left in the VNC of control flies 3 days after adult eclosion (Supplementary Figure 10C), but the total number of CCAP neurons in the VNC was larger after DIMM overexpression than in controls (Supplementary Figures 10B,D). Thus, Dimm appears to block the apoptotic process in a number of CCAP neurons. Interestingly, 60% of the adult flies with Dimm expression in CCAP neurons could not inflate their wings, and retained an inflated abdomen longer than control flies and flies after dInR expression alone (Supplementary Figures 10E,F). All the adult flies with Dimm/dInR expression displayed severe defects of wing inflation (Supplementary Figures 10E,F). CCAP acts together with co-localized bursicon in initiation of the ecdysis motor program (Park et al., 2003; Lahr et al., 2012). Targeted ablations of CCAP neurons resulted in death and severe failure for wing/abdominal inflation and cuticle tanning (Park et al., 2003). Our results indicate that also blocking apoptosis by Dimm disrupts functions of CCAP/Bursicon neurons required in ecdysis and postecdysis behavior.

EH is expressed in a pair of brain neuroendocrine cells in larval stages (Supplementary Figure 4A). These neurons send axon terminations to the corpora cardiaca of the ring gland and another branch extends medially along the length of the VNC. The EH neurons can also be detected in the pharate adult 8 h before ecdysis (Horodyski et al., 1993). After Dimm expression in these EH cells they are still detectable in 3–5 day old flies (Supplementary Figures 4B,C), but not in controls. The cell body size increased after Dimm/dInR expression (Supplementary Figure 4D). The axons extending to the ring gland in larvae was in adults seen terminating in the corpora cardiaca associated with the proventriculus, whereas the other axons ran to the end of the abdominal ganglion, similar to the morphology in wild type pharate adults (Horodyski et al., 1993) (Supplementary Figure 4C). However, the adult flies displayed normal eclosion behavior even with the continued presence of EH neurons caused by ectopic Dimm and Dimm/dInR expression.

In the larval SEG there are three pairs of leucokinin expressing SELK neurons (Supplementary Figure 1), whereas in the adult only one pair of SELKs can be detected (Figure 8D). It is not known whether the reduced number is caused by apoptosis, but ectopic expression of Dimm and Dimm/dInR results in flies with three pairs of SELKs (Figure 8D), suggesting that this might be the case, and that Dimm suppresses the cell death.

Larval PTTH producing neurons also survived well into adult life after Dimm, and Dimm/dInR expression resulted in increased cell body size and thicker axons and branches (Figure 9). In wild type *Drosophila* PTTH neurons were observed in the brain in late pupal stages, but could not be visualized after 3 d of adult life. After Dimm and Dimm/dInR expression the PTTH neurons could be seen in 3, 6, 12, and 37 d old adult flies. The cell body size and branch thickness grew significantly larger after Dimm/dInR expression compared to Dimm alone in 6 and 37 d old flies (Figures 9A,C). We also tested whether PTTH peptide is still produced in these adult neurons by applying antiserum to the peptide. Strong PTTH immunoreactivity was detected in 6 and 37 d adult flies (Figure 9B).

It has been shown that PTTH production is under transcriptional control by the circadian clock and PTTH release controls ecdysone secretion (McBrayer et al., 2007; Chen et al., 2014). In the larval brain branches from PTTH neurons are adjacent to dorsal branches of PDF-expressing clock neurons (Helfrich-Förster, 1998; Siegmund and Korge, 2001). Ecdysone in adult flies regulates sleep in a dose-dependent manner and mutants with disrupted ecdysone biosynthesis and receptor function displayed reduced sleep (Ishimoto and Kitamoto, 2010). PTTH, being a key regulator of ecdysone biosynthesis during development, may also affect ecdysone production in adult flies. After Dimm expression in PTTH neurons flies did not display any obvious change in developmental timing or body size. We monitored locomotor activity and sleep properties to investigate whether any effects were caused by Dimm expression in PTTH neurons (Supplementary Figure 11). The average locomotor activity during dark phase in 12L:12D conditions increased significantly after ectopic Dimm expression (Supplementary Figures 11A–D). The same phenotype was seen after dInR expression, but not during the light phase (Supplementary Figure 11E). In constant darkness conditions (DD), expression of Dimm alone caused a prominent increase of average locomotor activity but no changes were seen with either dInR or Dimm/dInR expression (Supplementary Figure 11F). The sleep pattern was slightly, but significantly, affected by dInR or/and Dimm expression in PTTH neurons (Supplementary Figures 11G–I). Either of dInR or Dimm expression resulted in decreased total sleep amount and sleep bout duration during the dark phase (Supplementary Figure 11I). Dimm alone also led to reduction of sleep bout duration and total sleep amount during light the phase compared to other genotypes (Supplementary Figure 11I). This result indicates that locomotor activity and sleep patterns were partly disrupted after Dimm and dInR expression in PTTH neurons, possibly as a result of ectopic interference with clock neurons, or due to altered ecdysone production. We did not detect any significant effect of our manipulations on circadian locomotor activity during constant darkness.

### Some direct targets of Dimm are upregulated after ectopic Dimm expression

Dimm overexpression in Dimm positive neuroendocrine cells (*c929*-Gal4 neurons) upregulates a range of transcriptional targets involved in pro-secretory and secretory pathways, neuropeptide biosynthesis, as well as other factors associated with a neuroendocrine phenotype (Park et al., 2008a, 2011; Hadžić et al., 2015). Here we monitored two direct Dimm targets, maelstrom and CAT-4 in Dimm negative PTTH neurons in third instar larval stage (Figure 10). Neither maelstrom nor CAT-4 is expressed in larval PTTH neurons in control flies. However, after Dimm and Dimm/dInR expression immunolabeling for both increased in the PTTH cell bodies (Figures 10A,B). This suggests that ectopic Dimm indeed upregulates known targets.

### DILPs from glia cells contribute to dInR mediated cell growth

**Figure 11:**
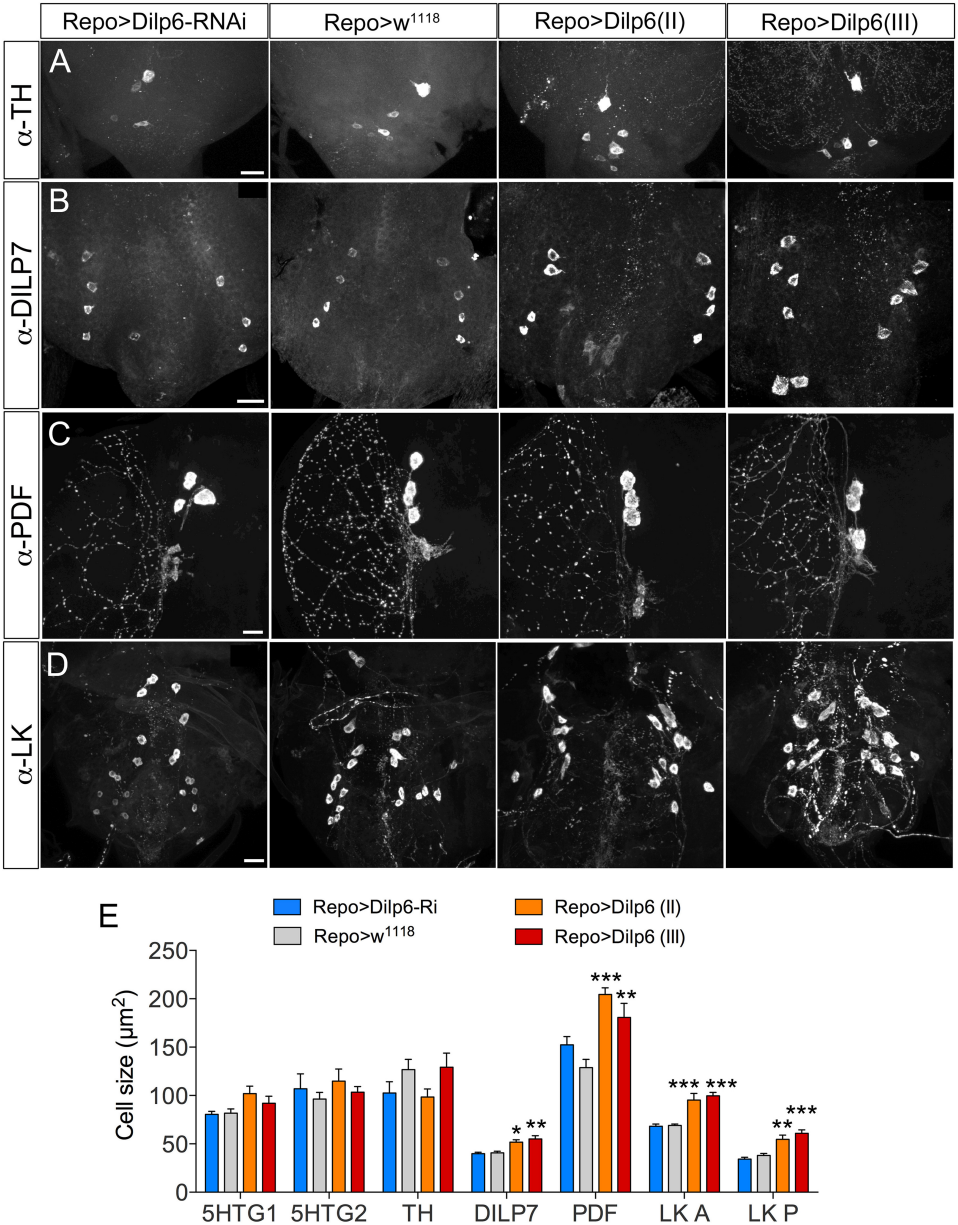
**DILP6 in glial cells is involved in dInR mediated cell growth in Dimm positive neurons**. The Repo-Gal4 driver was employed for targeted expression in glial cells. **(A)** Neither knockdown, nor overexpression of Dilp6 in glial cells has an effect on cell size of Dimm-negative dopaminergic neurons (TH-immunolabeled) in the VNC. **(B–D)** Dimm positive neurons display a significant growth after Dilp6 overexpression in glia cells. DILP7 expressing neurons in the adult VNC show increased cell body size after Dilp6 overexpression in glial cells **(B)**. The same phenotypes are observed in both PDF positive lLN_*v*_s **(C)** and leucokinin expressing ABLKs **(D)**. **(E)** Quantification of cell body sizes after manipulation of Dilp6 levels in glia cells using two different UAS-dilp6 lines (II and III). Only Dimm positive neuron types grow significantly and dilp6-RNAi has no effect. Data are presented as means ± S.E.M, *n* = 6–13 flies for each genotype from three crosses (^*^p < 0.05, ^**^p < 0.01, ^***^p < 0.001 as assessed by one way ANOVA followed by Dunnett's multiple comparisons test). Scale bar = 20 μm in **(A–D)**.

The additional increase of cell body size in both larvae and adults seen after coexpressing dInR and Dimm (compared to Dimm alone) indicates that the dInR and IIS plays a role in cell growth not only in the larval stage, but also in adults. What is the source of insulin-like peptide (DILP) that acts on ectopic dInR of the neuron types studied? Previous studies showed that inactivation of brain insulin producing cells (IPCs), or DILP7 expressing MP2 neurons, or altering DILP6 level in the fat body does not result in any cell size changes (Luo et al., 2013; Gu et al., 2014). Thus, some other DILP from a local niche may contribute to dInR mediated cell growth. Both DILP2 and DILP6 are expressed in subperineural glial cells of larvae, and are involved in regulation of nutrient dependent neuroblast reactivation (Chell and Brand, 2010; Sousa-Nunes et al., 2011; Hindle and Bainton, 2014; Schirmeier and Klämbt, 2015). We show here that GFP expression driven by *Dilp6*-Gal4 can be seen in marginal surface glia of the adult brain as well (Supplementary Figure 12), but we found no evidence for DILP2 expression in glial cells of the adult CNS. Since we had found dilp6 expression also in other glial cells (Luo et al., 2013), we increased the level of DILP6 by expressing UAS-*dilp6* more broadly under the control of *Repo*-Gal4, a line specific for glia cells (Figure 11). Cell body size of both Dimm positive and negative neurons in adult flies was monitored. After *dilp6* expression in glial cells we observed significantly larger cell bodies in selected Dimm positive neurons, such as DILP7 expression neurons, PDF expressing neurons (large LN_v_s) and ABLK neurons (Figures 11B–E). On the other hand, the cell body size of Dimm negative neurons was not affected by manipulation of DILP6 level in glia cells: two groups of serotonergic neurons in the T2 segment in the ventral nerve cord, and dopaminergic cells in the ventral nerve cord (Figures 11A,E). The organismal growth was unaffected by dilp6 manipulations in glial cells, which is consistent with a previous study (Chell and Brand, 2010).

## Discussion

Our study demonstrates that cell autonomous dInR-mediated growth in *Drosophila* occurs only in neurons naturally expressing Dimm (see also Luo et al., 2013) or after targeted ectopic expression of this transcription factor. Interestingly, we also found that ectopic Dimm alone can trigger neuron growth, and that co-expression of Dimm and dInR commonly results in further size increase of the targeted neurons. Thus, dInR-mediated growth seems to require upregulation of certain Dimm targets, and we propose that growth mediated by ectopic expression of Dimm alone could be due to an upregulation of native dInR, a known Dimm target (Hadžić et al., 2015). The additional growth of neurons, commonly seen after combined expression of Dimm and dInR (see Table 2), is likely due to an additive effect of the two ectopic proteins. It can also be noted that an earlier study showed that dInR-RNAi or Dimm-RNAi targeted to Dimm positive neurons lead to decreased size of their cell bodies (Luo et al., 2013).

Another finding is that the cellular response to ectopic Dimm and dInR expression varies among both the Dimm negative and Dimm positive neuron types, and to some extent depends on developmental stage. Thus, in larvae the cell body size of more than half of the tested neuron types was not affected by expressing Dimm alone (Table 1). When coexpressing Dimm together with dInR in Dimm positive 5 out of 7 types of neurons in larvae display increased size. However, in all 5 types of Dimm negative neurons tested, coexpression of Dimm and dInR results in increased cell size (Table 1). In adult flies, ectopic co-expression of Dimm and dInR promotes cell growth in all analyzed Dimm positive and Dimm negative neurons, ectopic Dimm alone gives rise to larger cell bodies in 11 out of 14 types of neurons (Table 2). Altogether we found that ectopic Dimm and dInR induces growth of peptidergic neuroendocrine cells and interneurons, two types of sensory cells, enteroendocrine cells, aminergic interneurons and glutamatergic motor neurons.

To determine the temporal effects of Dimm on cell growth, we performed conditional activation experiments on leucokinin-expressing neurons using the temperature-inducible Gal80 system. ABLKs did not respond to ectopic Dimm alone in larvae, whereas they enlarged when Dimm was targeted to adult neurons. However, in the adult stage cell growth did not occur in SELKs and LHLKs when Dimm was expressed conditionally. Thus, the effect of Dimm on size of SELKs and LHLKs observed in adults in non-conditional experiments probably occurs during metamorphosis.

An additional effect of ectopic Dimm was discovered here. We found that targeted Dimm expression leads to blocked apoptosis in five types of peptidergic neurons that normally undergo PCD during metamorphosis, or just after adult eclosion. These neurons that produce PTTH, leucokinin, corazonin, EH, and CCAP (Horodyski et al., 1993; Park et al., 2003; Choi et al., 2006; Lee et al., 2011; Veverytsa and Allan, 2012; Lee G. et al., 2013), remain detectable with peptide antisera several days into adulthood after targeted expression of Dimm (Table 2). Coexpression of Dimm and dInR in these neurons results in enlarged size of the surviving neurons.

We found that one likely source of ligand for dInR activation in Dimm positive neurons is DILP6 secreted from glial cells. This finding provides novel evidence of a local niche regulating neuron growth in developing and adult flies, similar to the DILP6-mediated growth and reactivation of quiescent neuroblasts in the early larva (Chell and Brand, 2010; Sousa-Nunes et al., 2011; Hindle and Bainton, 2014; Spéder and Brand, 2014).

Dimm has been established as a pro-secretory master regulator of properties essential for peptidergic neuroendocrine cells (Allan et al., 2005; Hewes et al., 2006; Park et al., 2008a,b; Park and Taghert, 2009; Hamanaka et al., 2010). Recently, a genome-wide identification of direct Dimm binding sites revealed a number of potential targets that are associated with neuroendocrine function, including secretory factors, Golgi trafficking, RNA metabolism, and large dense core vesicle related proteins (Hadžić et al., 2015). That study also shows that Dimm can regulate the dInR directly, which suggests that Dimm can control cell growth through the dInR-signaling pathway. This is supported by a decrease in cell size after knockdown of Dimm in leucokinin- (ABLK), DILP2-, and FMRFamide-immunoreactive neurons (Luo et al., 2013). Thus, in larvae Dimm-induced cell body growth of CCAP and sLNv neurons, and aCC motor neurons might be caused by its direct upregulation of the dInR. When monitoring neurons size in adults we found that most tested neuron types responded to Dimm by growth, suggesting that mechanisms differ during metamorphosis and later.

In addition to the function of Dimm as a master regulator of secretory capacity, it also acts as a part of a combinatorial transcriptional code for terminal differentiation of neurons (Hewes et al., 2003, 2006; Allan et al., 2005; Miguel-Aliaga et al., 2008). Thus, *Dimm* can cooperate with other transcription factors in a cascade to determine specification of neuron phenotypes. Therefore, the role of *Dimm* as part of a combinatorial code in cell specification also needs to be taken into account when interpreting our results. Different combinatorial codes contribute to specification of neuropeptidergic identities of neurons, and different expression levels of each code component are required for a given cell type (Herrero et al., 2003; Allan et al., 2005; Gauthier and Hewes, 2006; Benito-Sipos et al., 2010). One example of how ectopic Dimm may affect endogenous combinatorial codes in a cell-type specific fashion is in different subclasses of LK neurons. We find that growth of LK neurons in response to Dimm and Dimm/dInR expression varies depending on subclass both in larvae and adults. Gauthier and Hewes (2006) also showed that Dimm manipulations differentially affect LK neurons; the transcript level of *Lk* in *Dimm* mutants is slightly upregulated in LHLK and SELK neurons, but ABLKs show downregulated *Lk* transcription (Gauthier and Hewes, 2006). The LK peptide expression and terminal differentiation of the different types of LK neurons are known to depend on cell type-specific combinations of transcription factors. In the LHLK cells both *Squeeze* and *Apterous*, and a high level of either *Squeeze* or *Apterous* is sufficient to promote LK expression (Losada-Pérez et al., 2010), whereas in the SELK cells, only *Squeeze* is essential for LK expression, but *nab* is also involved in the specification of SELK cells (Losada-Pérez et al., 2010). Moreover, for specification in ABLK cells *Sqeeze* is dispensable, but the transcription factors *klumpfuss, nab*, and *castor* are required (Herrero et al., 2003, 2007; Benito-Sipos et al., 2010). Interestingly, in combination with other transcription factors, Dimm can induce ectopic peptide expression. For instance *Dimm* together with the temporal gene *grainy head* can trigger ectopic expression of FMRFamide peptide in ABLK neurons (Baumgardt et al., 2009; Benito-Sipos et al., 2010). We actually found that targeted expression of Dimm with the *c929*-Gal4 driver (representing Dimm neurons; Hewes et al., 2003) led to ectopic expression of ITP in a set of abdominal neurons, likely to produce CCAP (not shown). Considering these combined roles of Dimm and other transcription factors in specification of peptide expression and terminal differentiation, it is possible that ectopic Dimm in our experiments combines with endogenous transcription factors to produce a cell-specific effect. In this context, it is of interest that in larvae cell bodies of abdominal corazonin expressing neurons and serotonergic interneurons became smaller after Dimm expression. These two sets of neurons are derived from the same neuroblast lineage NB7-3 (Novotny et al., 2002; Lundell et al., 2003; Karcavich and Doe, 2005) and Numb/Notch signaling is involved in the specification of their cell fates (Lundell et al., 2003). There is no evidence that *dimm* acts directly on Numb/Notch signaling, but possibly targets of *Dimm* may interact with this signaling, and thereby lead to improper scaling of cell body size.

Our analysis of ectopic Dimm and dInR action included not only neuroendocrine cells, but also sensory cells, motor neurons, and gut endocrine cells. Two main types of sensory neurons were tested for ectopic Dimm and dInR expression: OSNs and retinal photoreceptors. The OSNs were targeted with the *Orco*-Gal4 driver, which covers about 70–80% of OSNs (Larsson et al., 2004). OSNs have axon terminations that synapse with local and projection neurons in the glomeruli of the antennal lobe (Masse et al., 2009; Rybak et al., 2016). Dimm and combined Dimm/dInR promote growth of both the DL3 glomerulus and entire antennal lobe. A portion of these OSNs, that supply axon terminations to at least 13 glomeruli (including DL3), express the neuropeptide sNPF (Carlsson et al., 2010), the sNPF receptor and dInR (Root et al., 2011), but in spite of their peptidergic nature they are likely to be Dimm negative. Possibly the endogenous expression of the dInR is why ectopic Dimm alone can influence growth of the axon terminations of these OSNs.

The effect of ectopic Dimm expression photoreceptor cells had been investigated previously using the same Gal4 drivers as in our study (Hamanaka et al., 2010), but without monitoring effects on cell size. We found that both Dimm and Dimm/dInR expression cause a severe distortion of eye development, including eye disc and optic lobe, during larval stages. Thus, ectopic Dimm alone not only changes the photoreceptors toward a peptidergic phenotype (Hamanaka et al., 2010), but also impedes the differentiation and arrangement of photoreceptor cells as well as the projection of axons into the optic lobe. Possibly, the loss of histaminergic signaling and altered synaptic structures of the photoreceptors (Hamanaka et al., 2010) cause a disrupted connectivity in the optic lobe. Nevertheless, we were able to reveal an increased axon diameter of R7/8 photoreceptors after ectopic dInR expression at the late pupal stage. Thus, photoreceptors, in spite of being Dimm negative, respond to dInR by growth, in contrast to CNS neurons.

The segmental motor neurons (aCC) in larvae are Dimm negative and their cell bodies grow after Dimm and Dimm/dInR expression. These neurons also respond to Dimm by changing the branching and bouton morphology in their axon terminations toward a peptidergic phenotype (unpublished data), similar to earlier findings using a different Gal4 driver (Bulgari et al., 2014). In addition we found that the aCC motor neurons displayed a loss of glutamate and synapse markers (unpublished data), similar to the loss of histaminergic phenotype of photoreceptors after ectopic Dimm expression (Hamanaka et al., 2010).

Finally, we investigated enteroendocrine (EE) cells of the midgut. A recent study reported that Dimm is not expressed in EE cells in the adult midgut under normal conditions, whereas Dimm expression starts in response to infection with Gram negative bacteria, *Pseudomonas entomophila* (Beebe et al., 2015). Ectopic dInR expression in TK expressing EE cells promotes a significant cell growth, and so does Dimm/dInR. Thus, like photoreceptors, the EE cells seem to be independent of Dimm for dInR-mediated growth, although it cannot be excluded that these cells express low levels of endogenous inducible Dimm. It is known that diet-stimulated midgut growth relies on DILP3 expression in the surrounding muscle (Amcheslavsky et al., 2014). Thus, this local source of DILP may be involved not only in triggering intestinal stem cell division, but also in regulation of EE cell growth.

We observed that several neuron types, which should have undergone PCD during metamorphosis or just thereafter, were persisting in the adult flies after ectopic Dimm expression. They were the abdominal corazonin expressing neurons (AbdCRZ) in female flies, CCAP neurons in subesophageal and abdominal ganglia, as well as PTTH and EH positive neurons in the brain. The PCD of AbdCRZ normally occurs within 6 h of the onset of metamorphosis, and is regulated by ecdysone signaling via the ecdysone receptor B and depend on the caspases *dronc* and *dark* (Choi et al., 2006). The *CCAP*-Gal4 by itself has been shown to suppress apoptosis of certain CCAP neurons in the ventral nerve cord, possibly by activating apoptosis suppressors, such as diap1 (Park et al., 2003; Lee G. G. et al., 2013). Thus, in our experiments we also observed a few of the CCAP neurons in the VNC remained in 6–7 day old control flies. However, expression of Dimm resulted in a larger number of cells expressing CCAP in the adult VNC than in controls. Importantly, four pairs of CCAP neurons in the SEG were present in adults after Dimm overexpression, but not in controls, suggesting blocked apoptosis.

The anti-apoptotic effect of Dimm may be due to a role in persistent maintenance of cell type identity in the adult fly. It has been reported that cellular differentiation is not only regulated during development, but also maintained in the adult, and requires a plasticity of transcription factor networks for long-term maintenance of subtype identity (Eade et al., 2012). The role of terminal differentiation genes and *Dimm* are thus persisting in the adult stage (see also Hewes et al., 2006). Conditional knock down of transcription factors in adult Tv neurons showed that *eya, ap, dac, dimm, grh*, and *Sqeeze* are all required for FMRFamide expression in adults (Eade et al., 2012). Thus, in our study, ectopic *Dimm* maybe becomes a key combinatorial factor for maintaining properties of the specific neuron types in adult flies and therefore PCD is overridden. On the other hand, it is also possible that certain *Dimm* targets interact with PCD-related genes and therefore block the apoptosis. An interesting outcome of the persistence of PTTH and CCAP neurons is that adult (PTTH) or posteclosion (CCAP) behavior were altered, maybe suggesting that these ectopic peptidergic neurons are capable of releasing peptides in the adult.

Finally, we investigated the possible source(s) of ligand activating the dInR to induce growth in mature neurons. It was shown previously that DILPs of the IPCs, as well as DILP7 and fat body-derived DILP6 are not responsible for dInR-mediated size scaling of neurons (Luo et al., 2013; Gu et al., 2014). We found here that expression of *dilp6* in glial cells increased the size in Dimm positive neurons, but not Dimm-negative ones. This suggests that DILP6 from the glial niche is one of the likely ligands important for dInR mediated cell growth also in late larval and adult flies. In early developing *Drosophila* larvae quite a few recent studies report that DILP2 and DILP6 expressed in blood brain barrier glia or DILP6 from underlying cortex glia are required for inducing growth and thus reactivation of proliferation in quiescent neuroblasts (Chell and Brand, 2010; Sousa-Nunes et al., 2011; Hindle and Bainton, 2014; Spéder and Brand, 2014). A nutrient signal from the fat body seems to activate the surface glia to trigger production and secretion of DILPs to reactivate neuroblasts (Chell and Brand, 2010; Rajan and Perrimon, 2012). It is likely that also nutrient signals are required for glial DILP6 release in later stages, since the Dimm-dependent dInR signaling is coupled to nutrient sensitive TOR signaling (Luo et al., 2013).

## Conclusions

Our study reports a comprehensive screen of the effects of ectopic Dimm and dInR on growth and differentiation of a broad set of neuron types, including neuroendocrine cells, enteroendocrine cells, sensory cells, interneurons, and motor neurons. We confirmed that dInR mediated neuron growth occurs in a Dimm dependent manner. Expressing both Dimm and dInR in Dimm negative neurons induced cell growth, whereas dInR alone did not. On the other hand we found that Dimm alone can induce cell growth in certain cell types during metamorphosis or in the adult stage. This effect is likely to be associated with the function of Dimm as part of a combinatorial code for terminal cell differentiation. In other neuron types ectopic Dimm may act in continued maintenance of neuron identity in the adult CNS and therefore inhibit apoptosis of neurons destined for PCD. Taken together our results suggest that Dimm plays different roles during different developmental stages and in different neuron types. We also showed that DILP6 from glia cells is one likely source of ligand for dInR-mediated neuron growth in the CNS.

## Author contributions

Designed study: YL, JL, DN. Performed experiments: YL, JL. Interpreted data: YL, JL, DN. Wrote paper: YL, DN. Supervised study: DN. Obtained funding: DN. All authors edited and approved the paper.

### Conflict of interest statement

The authors declare that the research was conducted in the absence of any commercial or financial relationships that could be construed as a potential conflict of interest.
