# Supplementary material for: The Drosophila Transcription Factor Dimmed Affects Neuronal Growth and Differentiation in Multiple Ways Depending on Neuron Type and Developmental Stage
Source: Front Mol Neurosci. 2016 Oct 13;9:97. doi: 10.3389/fnmol.2016.00097 (PMC5064288; doi:10.3389/fnmol.2016.00097)
Supplement: Supplementary file 1 [file Image1.PDF]

Supplementary Material

The *Drosophila* transcription factor Dimmed affects neuronal growth and differentiation in multiple ways depending on neuron type and developmental stage

Yiting Liu, Jiangnan Luo, Dick R Nässel*

*** Correspondence:** Corresponding Author: dnassel@zoologi.su.se

1 Supplementary Figures

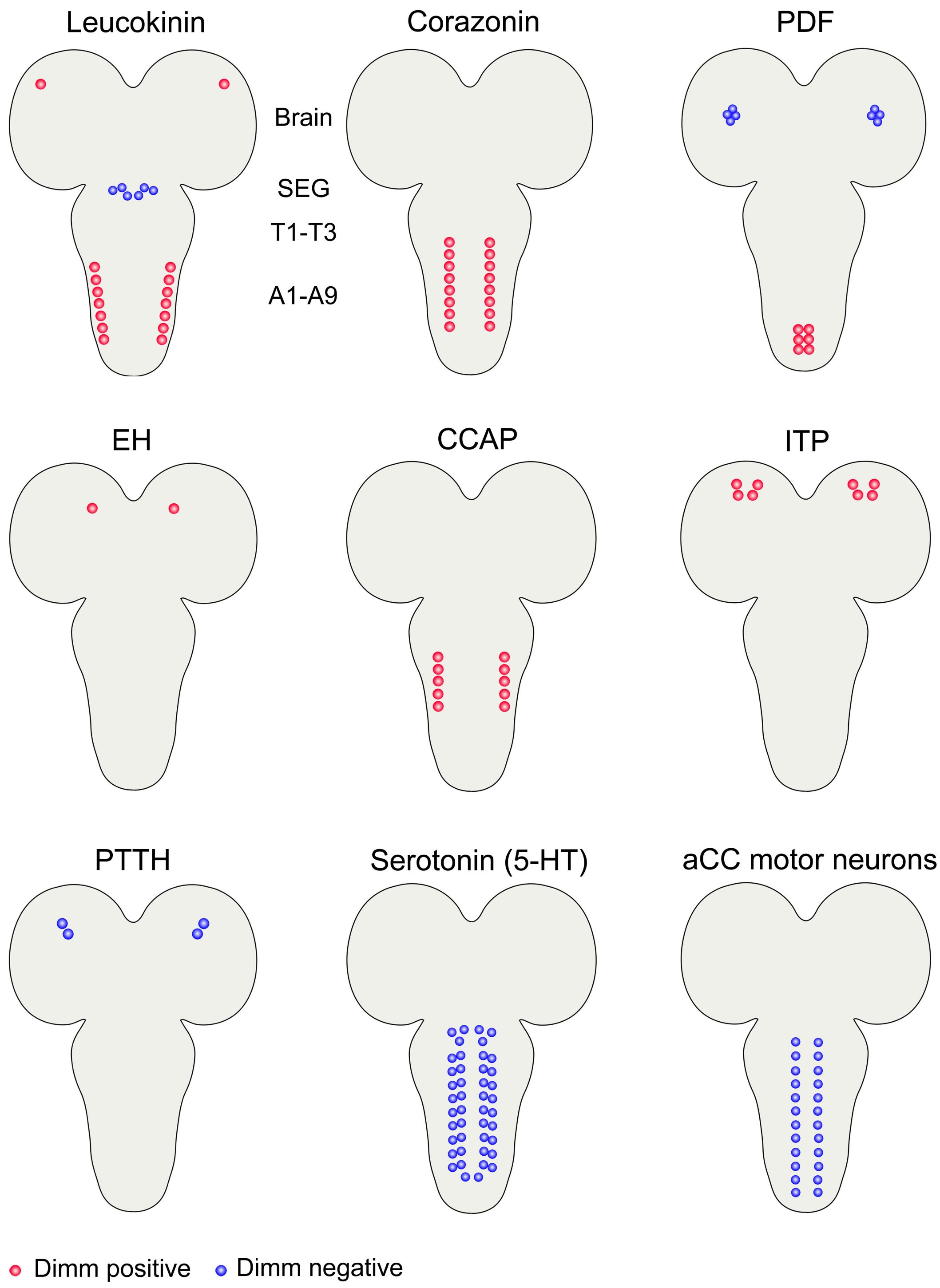

Supplementary Figure 1. Distribution of Dimm positive and negative neurons in the CNS of third instar larvae that were studied. SEG, subesophageal ganglion; T1-T3, thoracic neuromeres 1-3; A1-A9, abdominal neuromeres 1-9.

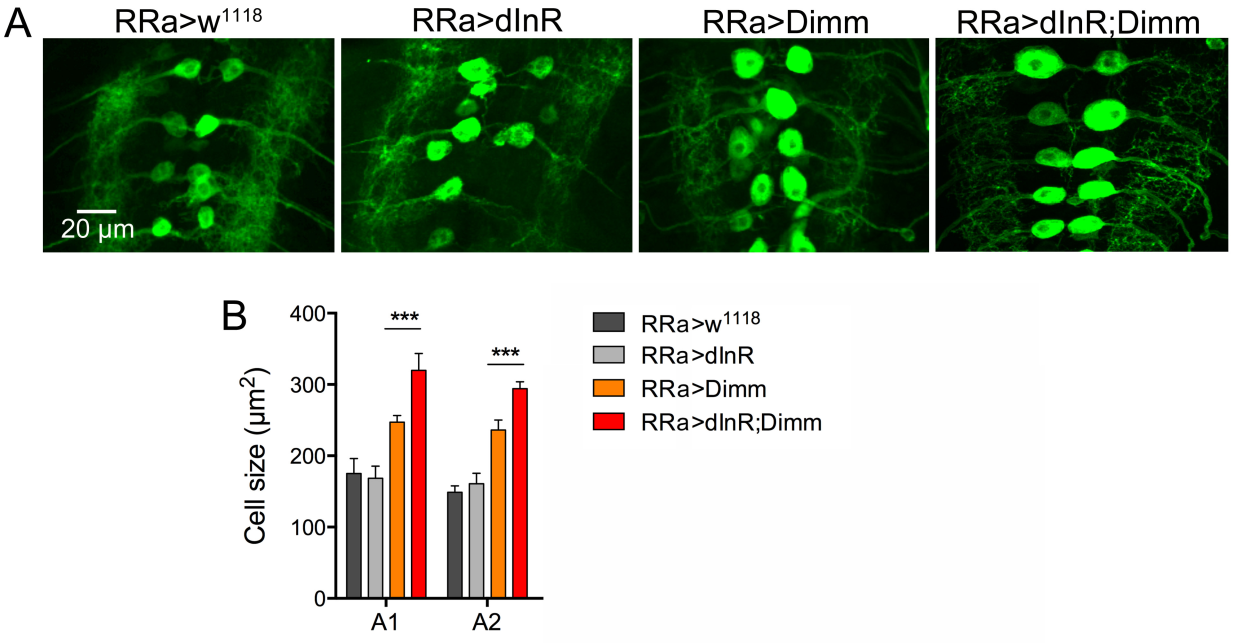

Supplementary Figure 2. Segmental aCC motor neurons in abdominal ganglia of third instar larvae are Dimm negative and grow after ectopic Dimm and Dimm/dInR expression. **A**. GFP expression in RRa-Gal4 neurons (RRa;mCD8-GFP-Gal4) reveal that Dimm and Dimm/dInR induces growth of aCC cell bodies. B. Quantification of cell body sizes in different genotypes. Data are presented as means ± S.E.M, n = 9-12 flies for each genotype from three crosses (***$p<0.001$ as assessed by unpaired Students' *t*-test).

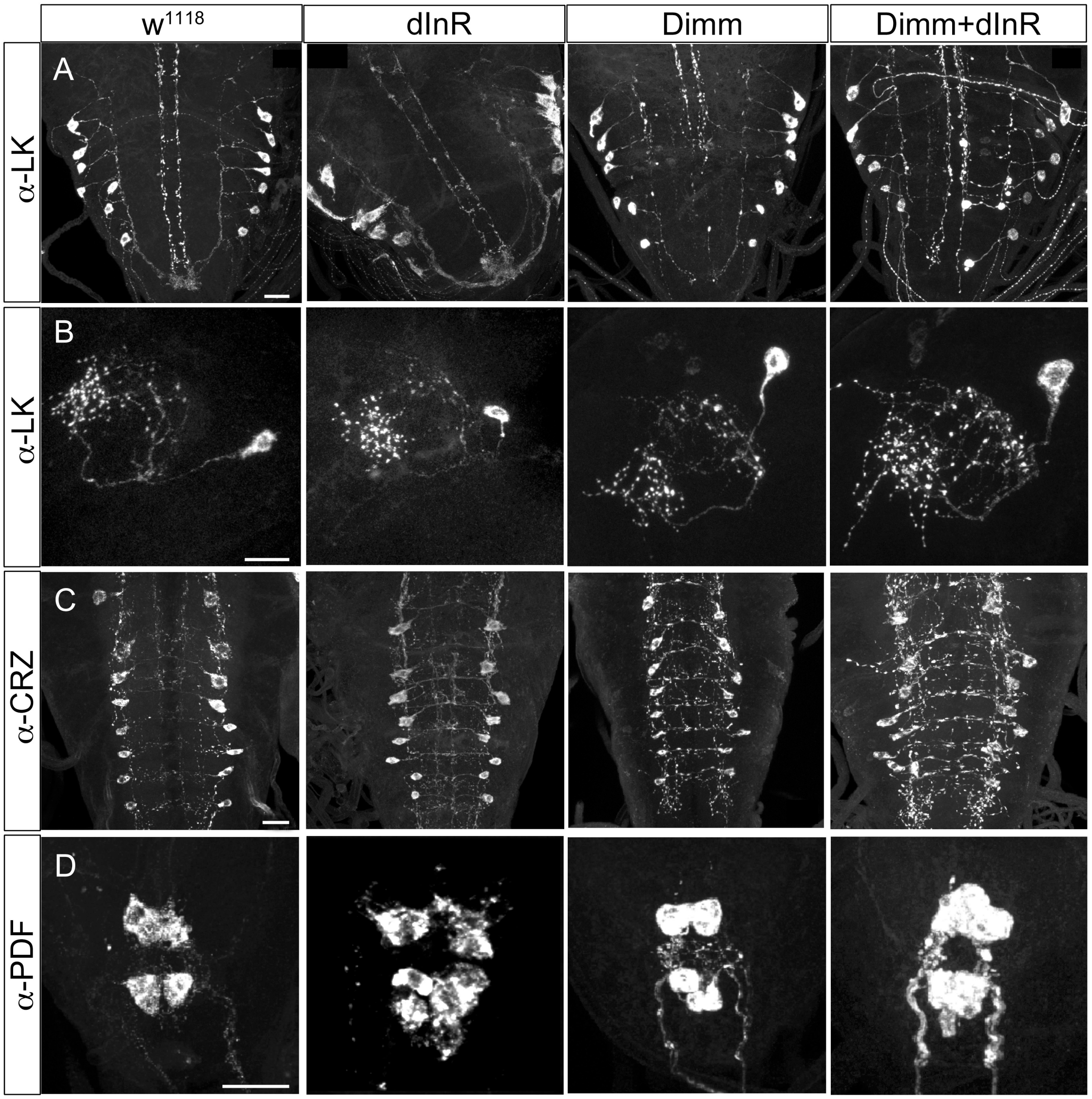

Supplementary Figure 3. Ectopic expression of dInR, Dimm and Dimm/dInR in Dimm positive neurons in the third instar larva. **A-B**. Leucokinin (anti-LK) expressing neurons in the abdominal ganglion (ABLKs) and lateral horn of larval brain (LHLKs) are Dimm positive. Cell bodies of both types of LK neurons enlarge after Dimm/dInR expression but are not affected by Dimm alone. **C**. Corazonin expressing neurons in the VNC (vCrz) display a decreased cell body size after Dimm expression, whereas after expressing dInR or Dimm/dInR larger cell bodies are observed. **D**. Dimm/dInR as well as dInR alone induces growth of PDF expressing neurons in the VNC. There is no significant change in cell body size after Dimm expression alone. Scale bar = 20μm in **A** - **D**.

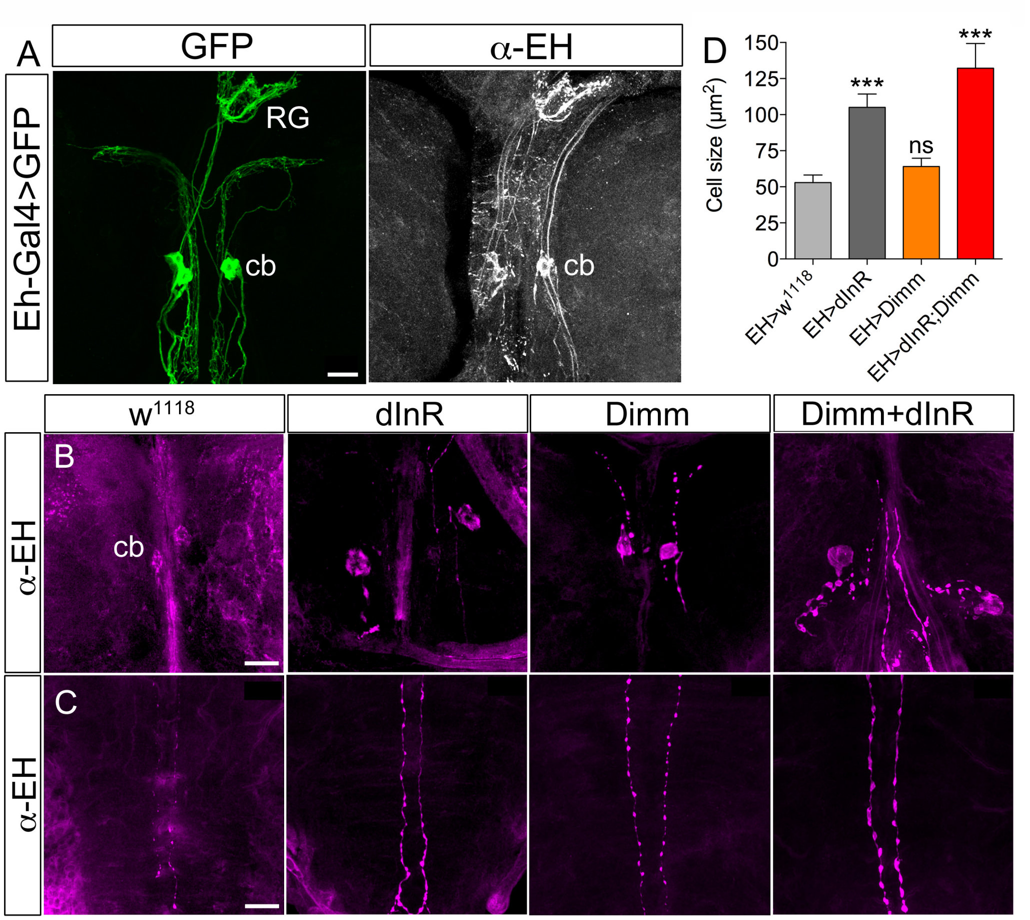

Supplementary Figure 4. Dimm positive eclosion hormone (EH) expressing cells are affected by ectopic expression of Dimm and dInR. **A**. In the third instar larvae, one pair of EH cells is located in the median brain. Anti EH (white) immunolabeling is colocalized with GFP expression (green). RG, ring gland; cb; cell body. **B**. Expression of dInR with an Eh-Gal-4 driver promotes growth of EH cells and Dimm/dInR enhances this effect further. Dimm alone does not affect cell body size. **C**. EH positive cells send axon projections along the midline of brain and VNC. The varicosities of axon processes enlarge after Dimm/dInR expression. **D**. Quantification of cell body sizes after ectopic expression of dInR, Dimm or Dimm/dInR compared to controls. Data are presented as means ± S.E.M, n = 9-10 flies for each genotype from three crosses (***$p<0.001$ ns - not significant as assessed by unpaired Students' *t*-test). Scale bar = 20μm in **A** – **C.**

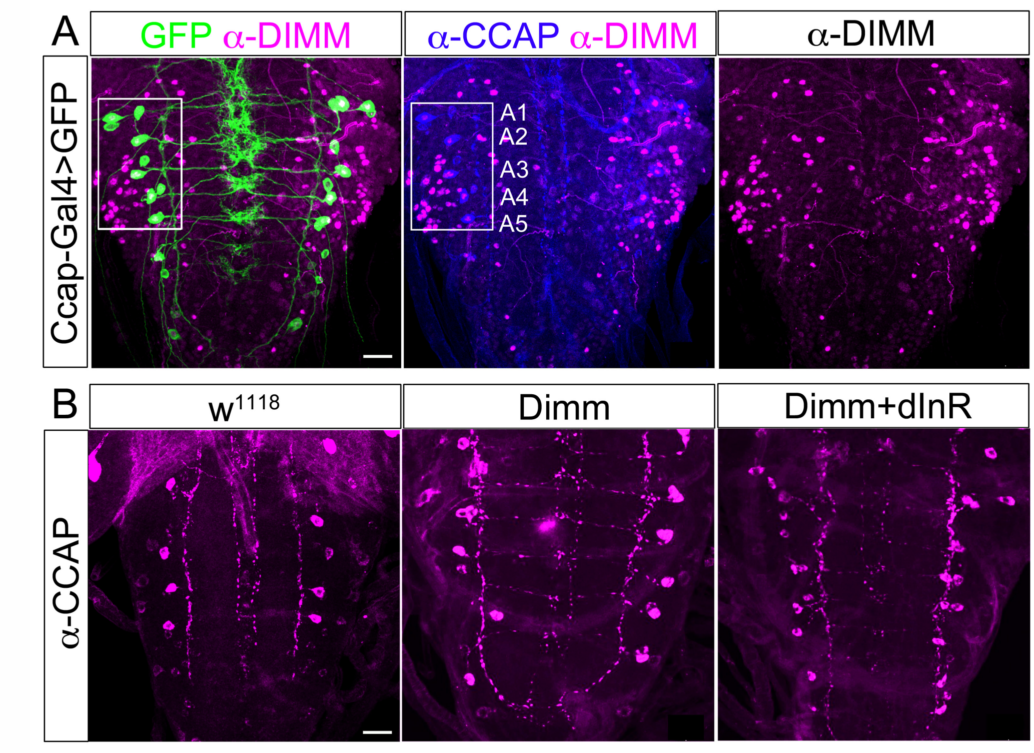

Supplementary Figure 5. Larval cardioactive peptide (CCAP) producing neurons express Dimm and yet they increase in size after ectopic expression of Dimm. **A**. Neurons in the larval ventral nerve cord expressing Ccap-Gal4>mCD8-GFP are labeled with anti-Dimm (magenta), anti-CCAP (blue) and anti-GFP (green). The efferent CCAP neurons (EN-CCAP) in the abdominal neuromeres A1-A5 are strongly stained by anti CCAP. High level of Dimm expression is seen in these neurons (in the white box). The CCAP interneurons display weaker CCAP immunofluorescence and low level of Dimm expression. **B**. Ectopic Dimm expression leads to growth of cell bodies of EN-CCAPs, but there is no effect of Dimm/dInR expression.

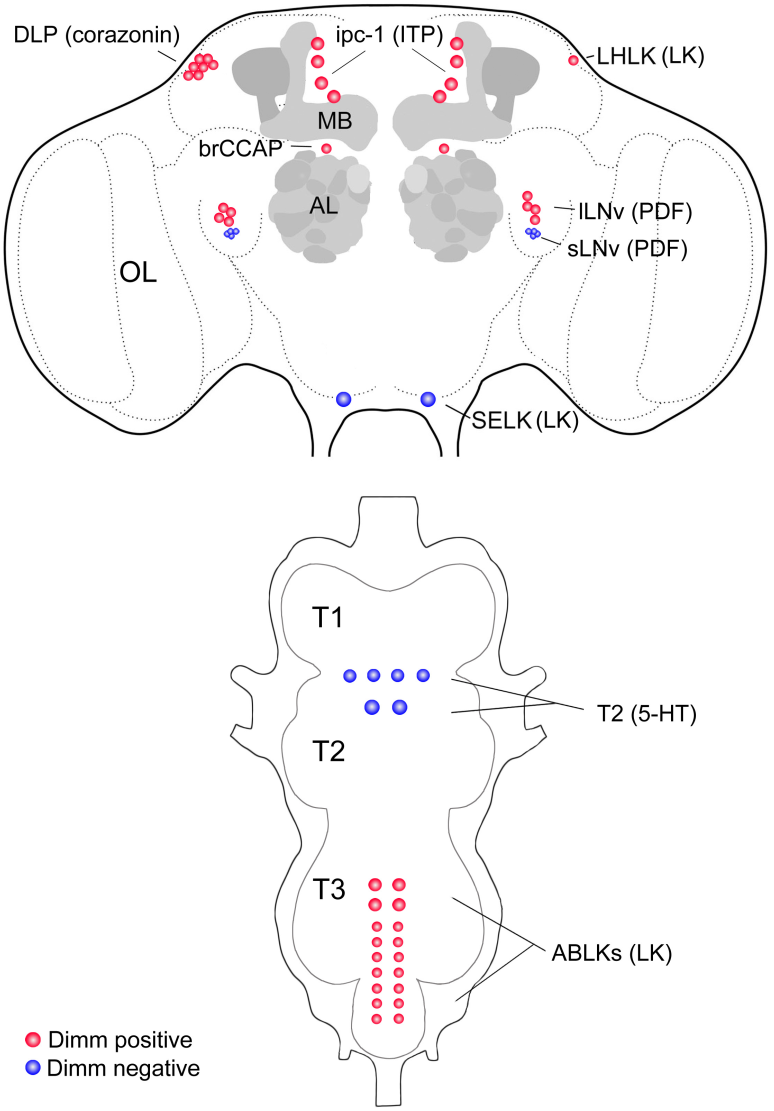

Supplementary Figure 6. Schematic distribution of Dimm positive and negative neurons that were studied in the adult CNS. Note that the DLP and LHLK neurons are expressed in both hemispheres (for simplicity shown on one side only). LK, leucokinin; ITP, ion transport peptide; PDF, pigment-dispersing factor; 5-HT, serotonin; OL, optic lobe; MB, mushroom body; AL, antennal lobe; T1-T3, thoracic neuromeres (the abdominal neuromeres are located where the ABLKs are depicted).

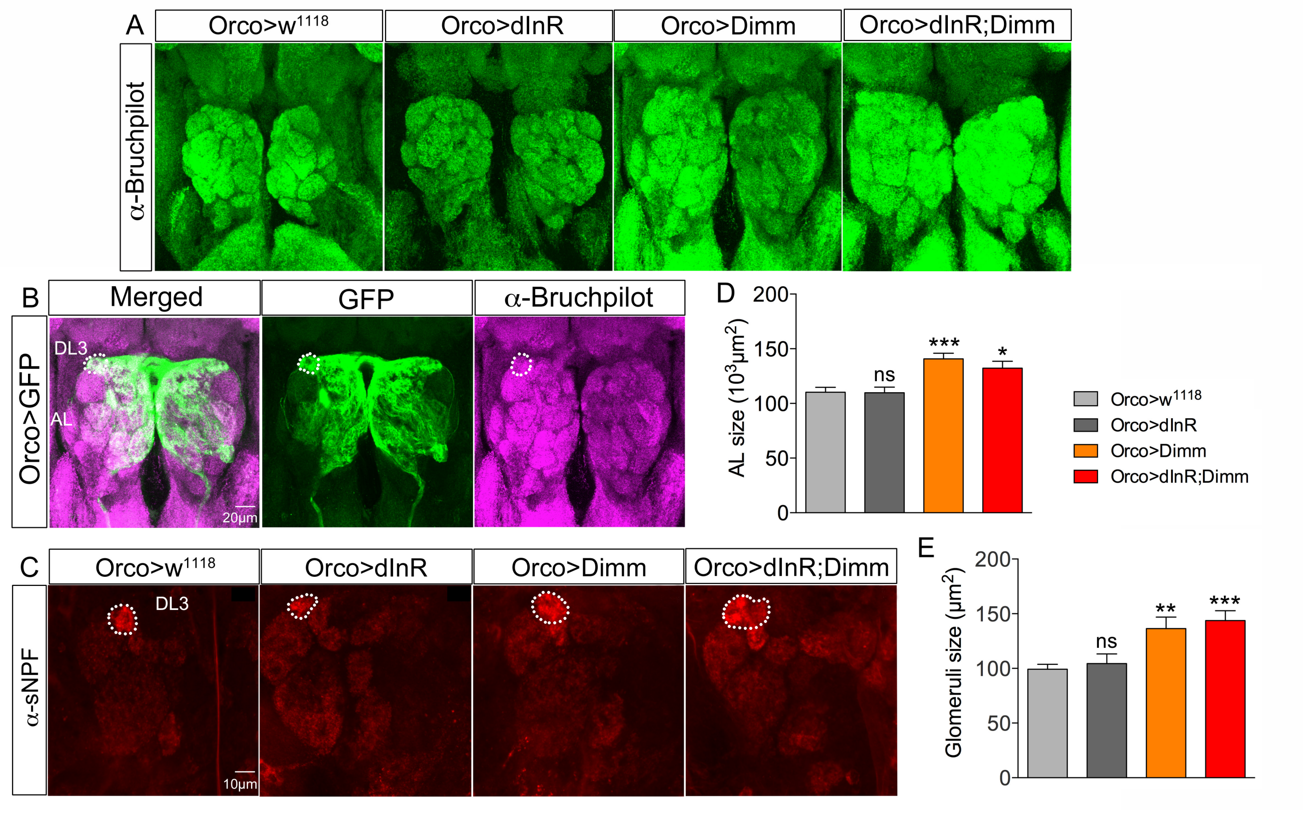

Supplementary Figure 7. Ectopic expression of Dimm and Dimm/dInR induces growth in olfactory sensory neurons (OSNs) in the adult brain. **A**. The antennal lobe was labeled with antibody to the synapse protein Bruchpilot. The size of antennal lobes increased after Dimm and Dimm/dInR expression. **B**. Orco-Gal4 driven GFP is expressed in OSNs (green) innervating the antennal lobe (anti-Bruchpilot, magenta). **C**. The dorsal lateral 3 (DL3) glomerulus in the dashed circle is stained by anti-sNPF (red) and its size was measured after dInR, Dimm and Dimm/dInR expression compared to control. Both Dimm alone and Dimm/dInR increase the size of DL3 glomerulus, but dInR alone does not. **D-E**. Quantification of size changes after ectopic expression of dInR, Dimm or Dimm/dInR compared to controls. In **C** the whole antennal lobe (AL) was measured and in **D** the DL3 glomerulus. Data are presented as means ± S.E.M, n = 8-15 flies for each genotype from three independent crosses (*p<0.05, **p<0.01, *** p<0.001 ns - not significant as assessed by unpaired Students' *t*-test).

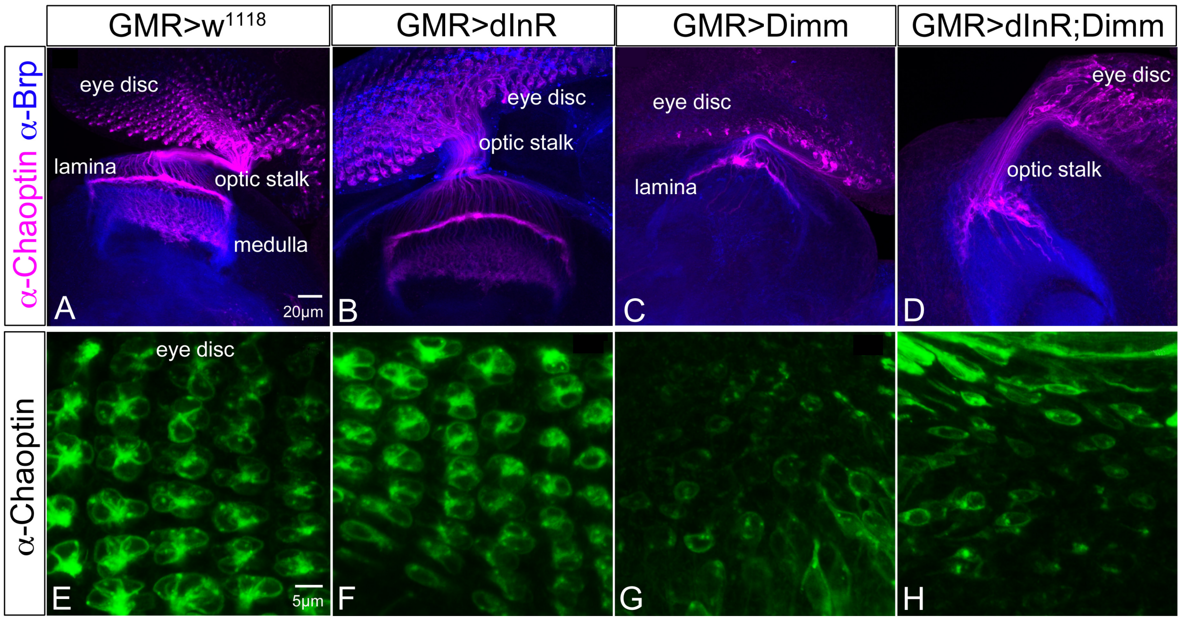

Supplementary Figure 8. Effects of ectopic expression of dInR, Dimm and Dimm/dInR in developing photoreceptor cells in third instar larvae. **A**. Photoreceptors in ommatidial clusters in the eye disc send axonal projections through the optic stalk into the developing lamina and medulla in the prospective optic lobe. Photoreceptor cells are labeled by anti Chaoptin (magenta), neuropil with antiserum to the synapse protein Bruchpilot (Brp; blue). **B-D**. Ectopic expression of Dimm and Dimm/dInR, but not dInR, leads to a distorted arrangement of photoreceptor cells in the eye disc, and their axon terminations fail to form a normal pattern in the developing medulla. **E.** Differentiating photoreceptors display a hexagonal array of the normal eye disc marked with anti Chaoptin (green). **F-H**. Photoreceptor cells do not form a normal hexagonal array in the eye disc after Dimm and Dimm/dInR expression, but are not affected by dInR.

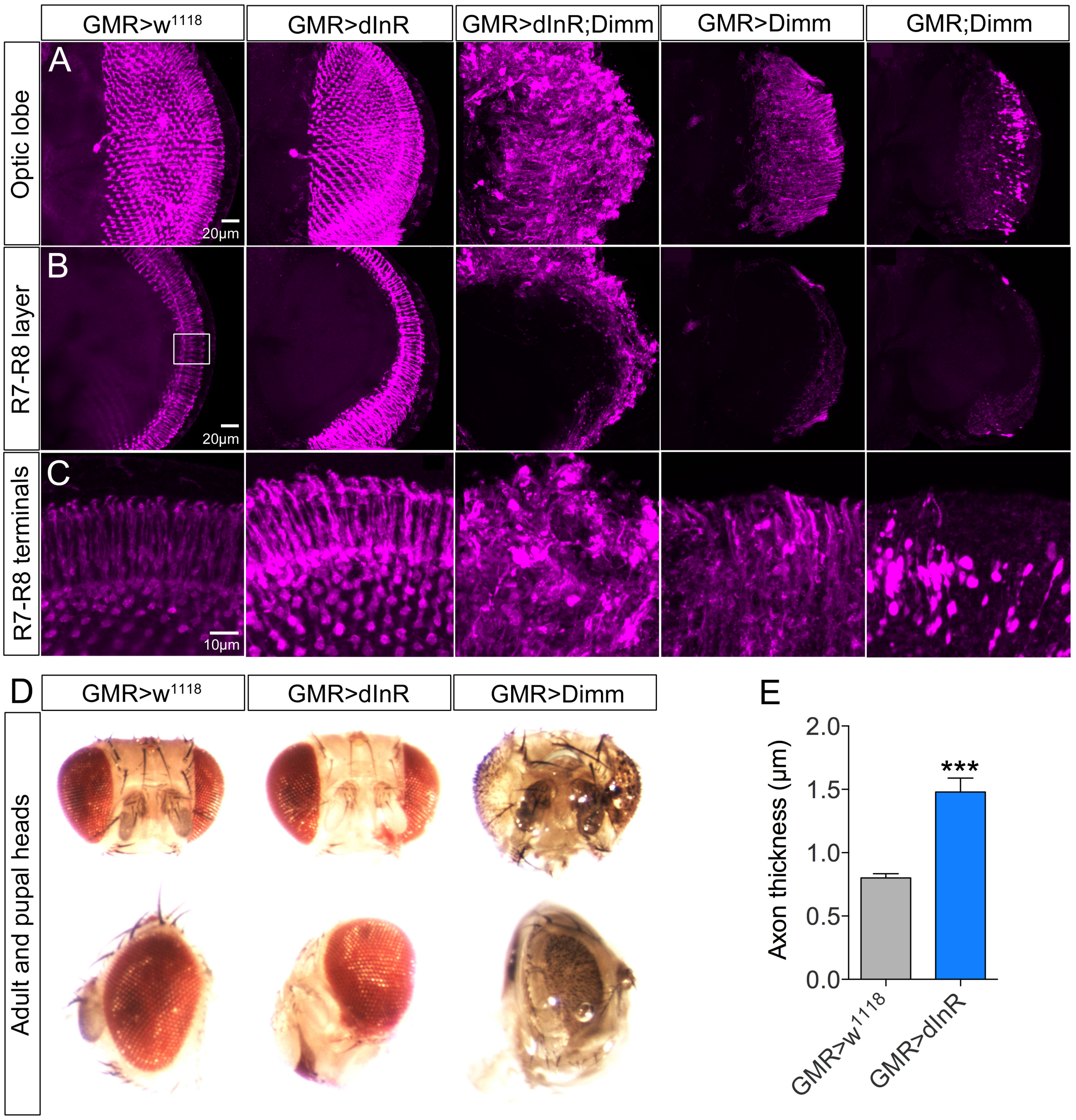

Supplementary Figure 9. Ectopic expression of Dimm and Dimm/dInR in photoreceptor cells causes disrupted eye development and lethality in the pupal stage, whereas dInR induces cell growth. **A-C**. Optic lobes from pharate adults were stained with anti Chaoptin (magenta). The axons of R7 and R8 photoreceptor cells form medulla columns and terminate in the M6 and M3 strata (boxed in **B**). The diameters (cross section) of axon terminations in the medulla were monitored after experiments (as seen in **C**). Ectopic Dimm and Dimm/dInR expression drastically disrupts the columnar pattern of R7 and R8 axon terminations (**C**). Here we also utilized a recombinant line of GMR-Gal4; UAS-Dimm (GMR;Dimm). This fly line is able to develop into the adult stage, whereas direct Dimm expression under the control of GMR-Gal4 leads to lethality in pupae. **D**. Heads of pharate adult flies after ectopic Dimm expression, compared to those of adult control flies (GMR>w^{1118}) and after dInR expression. Compound eyes of Dimm expressing flies display reduced size and improper eye differentiation. **E**. Thickness of axon terminations in controls and dInR expressing flies. Data are presented as means ± S.E.M, n = 7-8 flies for each genotype from three independent crosses (***$p<0.001$ as assessed by unpaired Students' *t*-test). Scale bar = 20µm in **A** - **B**, 10µm in **C**.

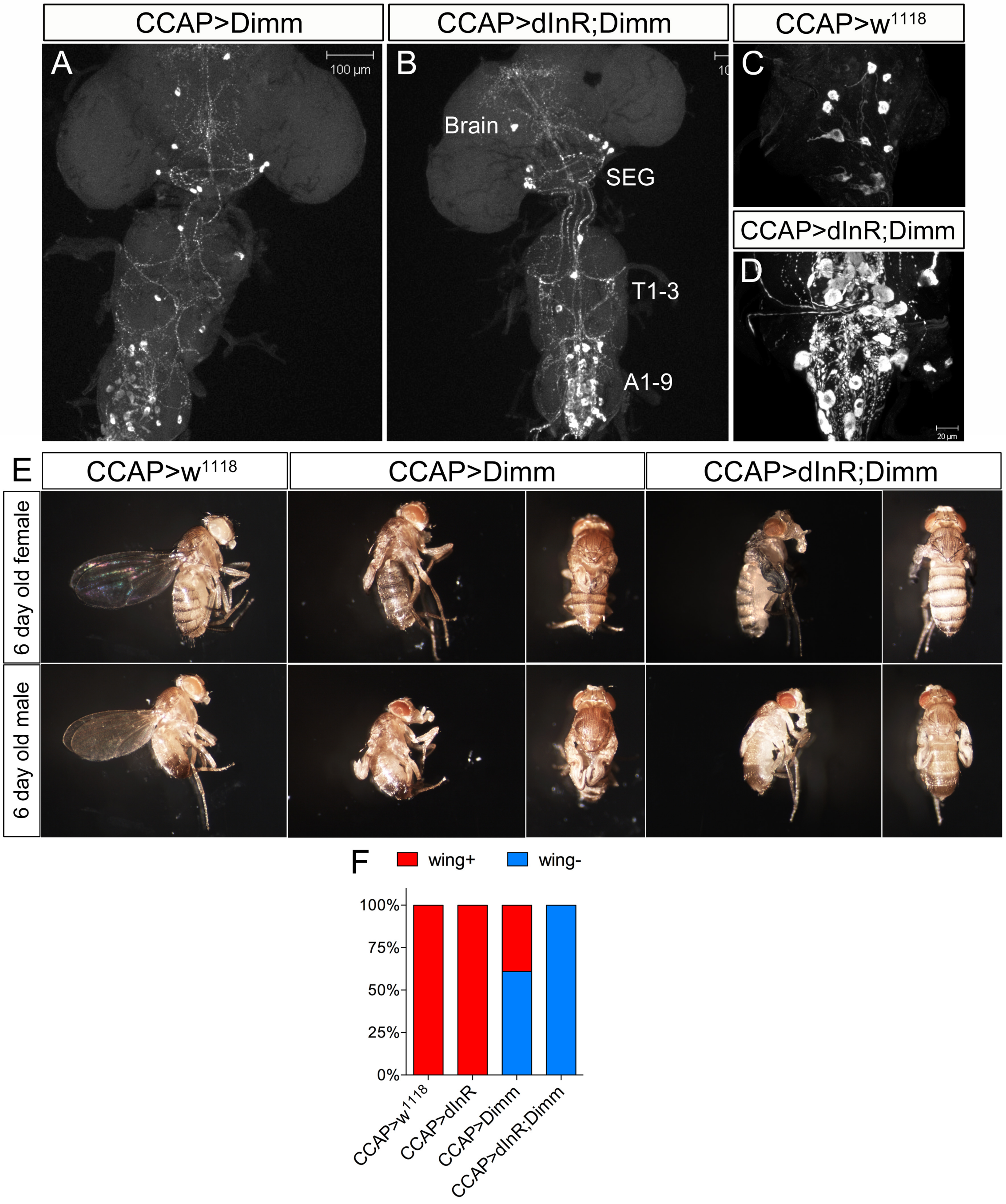

Supplementary Figure 10. The CCAP expressing neurons in subesophageal, thoracic and abdominal ganglion do not undergo programmed apoptosis after Dimm and Dimm/dInR expression and this affects wing expansion. **A-B**. CCAP neurons in the CNS of 6 d old flies after ectopic Dimm or Dimm;dInR expression. The CCAP neurons of the subesophageal ganglion (SEG) are absent in controls (shown in Fig. 10E). **C-D.** In the abdominal ganglia a few CCAP neurons survive apoptosis also in controls (C), but with ectopic Dimm or Dimm/dInR the full number of larval neurons are still seen, and they grow (D). **E**. Six day old male and female flies were monitored for wing expansion. After Dimm expression most of flies fail to expand their wings, and with Dimm/dInR expression no flies are able to perform wing inflation and the tanning of body is weaker than in control flies. **F**. Percentage of flies displaying wing expansion after dInR, Dimm and Dimm/dInR expression compared to controls.

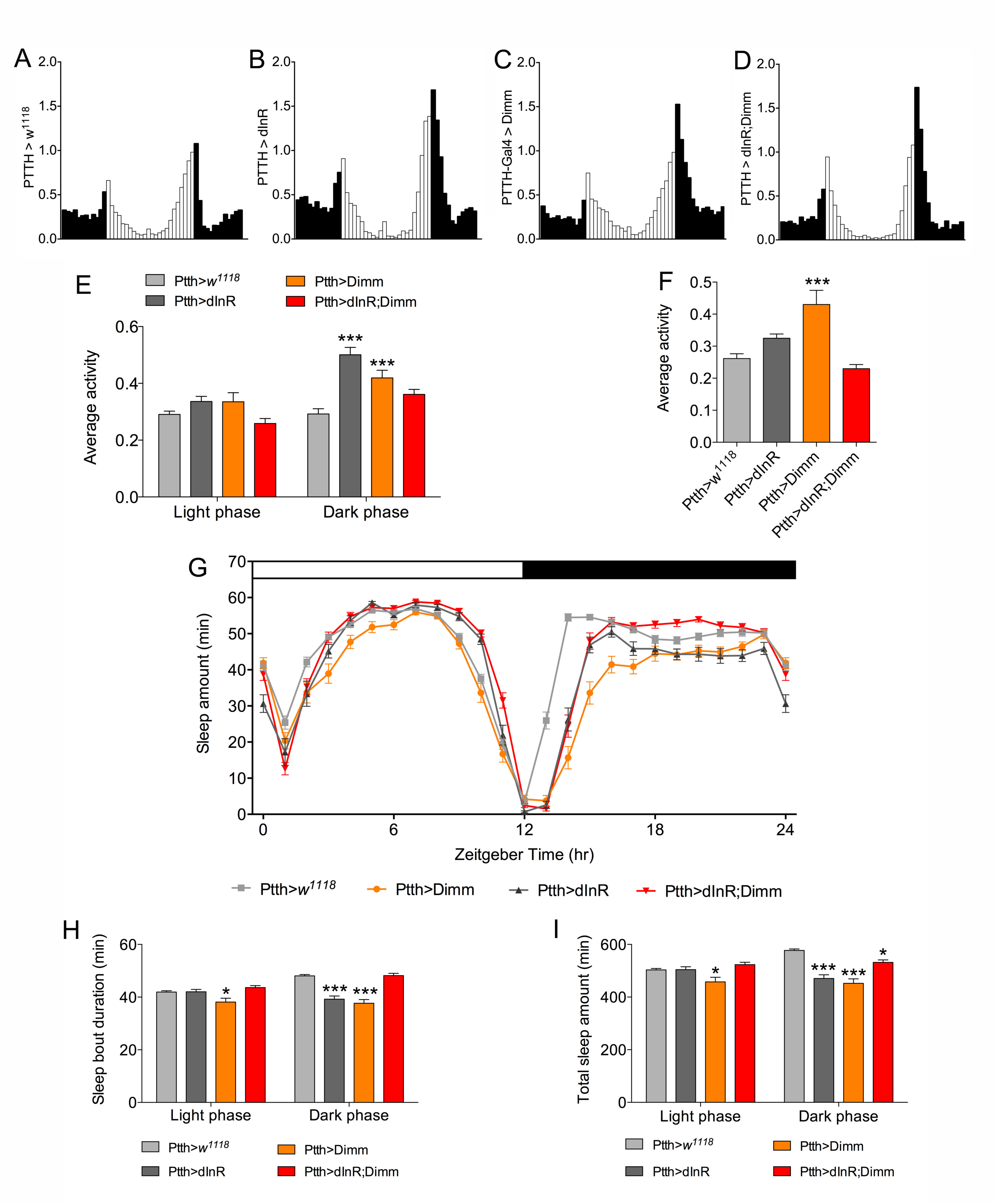

Supplementary Figure 11. Locomotor activity and sleep under 12L:12D conditions were affected by ectopic dInR, Dimm and Dimm/dInR in PTTH neurons. Activity was recorded in flies of different genotypes for 10 days. **A – D.** Actograms showing average locomotor activity of the four genotypes

monitored over 5 days of 12L:12D. The quantification of the data are shown in panel E. **E.** There is no significant difference in average activity during light phase, whereas Dimm and/or dInR expression in PTTH neurons leads to a significant increase of average activity during dark phase. **F.** Average locomotor activity was analyzed in constant darkness (DD) and flies exhibit an increased average activity after Dimm or dInR expression, but Dimm/dInR has no effect. **G**. Sleep pattern after Dimm and Dimm/dInR expression in PTTH neurons, compared to controls and dInR expression. The X-axis represents the 12L:12D conditions. During zeitgeber time ZT1 –ZT12 light is on, ZT13-ZT24 is light off. Each point represents sleep amount (min) within each hour. **H**. Sleep bout duration during light phase shows a significant decrease only after Dimm expression. Dimm or dInR expression alone leads to a decrease of sleep amount during dark phase. **I**. The same trend is also seen in total sleep amount. Besides, Dimm/dInR displayed a slight but significant sleep decrease compared to control. Data are presented as means ± S.E.M, n = 29-32 flies for each genotype from three crosses (*p<0.05, *** p<0.001 as assessed by unpaired Students' *t*-test).

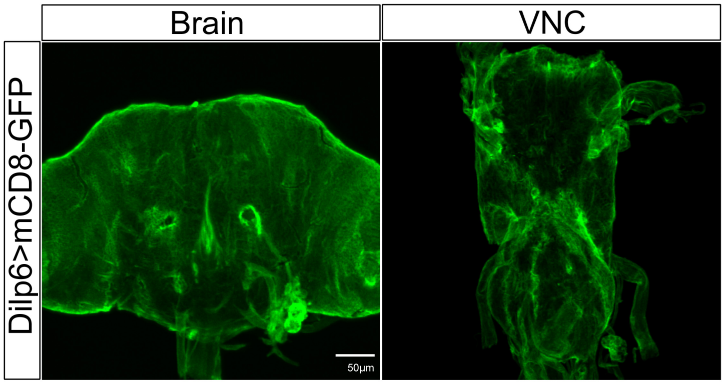

Supplementary Figure 12. DILP6 expression in the marginal surface of the adult brain and ventral nerve cord (VNC). GFP expression driven by dilp6-Gal4 indicated the possible location of dilp6 expressing glial cells in the surface of the adult CNS.
